# Examining Individual and Synergistic Contributions of PTSD and Genetics to Blood Pressure: A Trans-Ethnic Meta-Analysis

**DOI:** 10.3389/fnins.2021.678503

**Published:** 2021-06-23

**Authors:** Jennifer A. Sumner, Adam X. Maihofer, Vasiliki Michopoulos, Alex O. Rothbaum, Lynn M. Almli, Ole A. Andreassen, Allison E. Ashley-Koch, Dewleen G. Baker, Jean C. Beckham, Bekh Bradley, Gerome Breen, Jonathan R. I. Coleman, Anders M. Dale, Michelle F. Dennis, Norah C. Feeny, Carol E. Franz, Melanie E. Garrett, Charles F. Gillespie, Guia Guffanti, Michael A. Hauser, Sian M. J. Hemmings, Tanja Jovanovic, Nathan A. Kimbrel, William S. Kremen, Bruce R. Lawford, Mark W. Logue, Adriana Lori, Michael J. Lyons, Jessica Maples-Keller, Matig R. Mavissakalian, Regina E. McGlinchey, Divya Mehta, Rebecca Mellor, William Milberg, Mark W. Miller, Charles Phillip Morris, Matthew S. Panizzon, Kerry J. Ressler, Victoria B. Risbrough, Barbara O. Rothbaum, Peter Roy-Byrne, Soraya Seedat, Alicia K. Smith, Jennifer S. Stevens, Leigh Luella van den Heuvel, Joanne Voisey, Ross McD Young, Lori A. Zoellner, Caroline M. Nievergelt, Erika J. Wolf

**Affiliations:** ^1^Department of Psychology, University of California, Los Angeles, Los Angeles, CA, United States; ^2^Department of Psychiatry, University of California, San Diego, San Diego, CA, United States; ^3^Veterans Affairs San Diego Healthcare System, VA Center of Excellence for Stress and Mental Health (CESAMH), San Diego, CA, United States; ^4^Department of Psychiatry and Behavioral Sciences, Emory University, Atlanta, GA, United States; ^5^Yerkes National Primate Research Center, Atlanta, GA, United States; ^6^Department of Psychological Sciences, Case Western Reserve University, Cleveland, OH, United States; ^7^National Center on Birth Defects and Developmental Disabilities, Centers for Disease Control and Prevention, Atlanta, GA, United States; ^8^NORMENT, Institute of Clinical Medicine, University of Oslo, Oslo, Norway; ^9^Division of Mental Health and Addiction, Oslo University Hospital, Oslo, Norway; ^10^Duke Molecular Physiology Institute, Duke University, Durham, NC, United States; ^11^Durham VA Health Care System, Durham, NC, United States; ^12^Department of Psychiatry and Behavioral Sciences, Duke University School of Medicine, Durham, NC, United States; ^13^VA Mid-Atlantic Mental Illness Research, Education, and Clinical Center (MIRECC), Genetics Research Laboratory, Durham, NC, United States; ^14^Atlanta VA Health Care System, Decatur, GA, United States; ^15^Genetic and Developmental Psychiatry Centre, Institute of Psychiatry, Psychology, and Neuroscience, King’s College London, London, United Kingdom; ^16^NIHR BRC at the Maudsley, King’s College London, London, United Kingdom; ^17^Department of Radiology, University of California, San Diego, San Diego, CA, United States; ^18^Department of Neurosciences, University of California, San Diego, San Diego, CA, United States; ^19^Department of Psychiatry, Harvard Medical School, Boston, MA, United States; ^20^McLean Hospital, Belmont, MA, United States; ^21^Department of Psychiatry, Faculty of Medicine and Health Sciences, Stellenbosch University, Cape Town, South Africa; ^22^South African Medical Research Council/Stellenbosch University Genomics of Brain Disorders Research Unit, Faculty of Medicine & Health Sciences, Stellenbosch University, Cape Town, South Africa; ^23^Department of Psychiatry and Behavioral Neurosciences, School of Medicine, Wayne State University, Detroit, MI, United States; ^24^School of Biomedical Sciences, Queensland University of Technology, Kelvin Grove, QLD, Australia; ^25^National Center for PTSD, Behavioral Science Division, VA Boston Healthcare System, Boston, MA, United States; ^26^Department of Psychiatry, Boston University School of Medicine, Boston, MA, United States; ^27^Department of Biostatistics, Boston University School of Public Health, Boston, MA, United States; ^28^Biomedical Genetics, Boston University School of Medicine, Boston, MA, United States; ^29^Department of Gynecology and Obstetrics, Emory University, Atlanta, GA, United States; ^30^Department of Psychological & Brain Sciences, Boston University, Boston, MA, United States; ^31^Department of Psychiatry, Case Western Reserve University, Cleveland, OH, United States; ^32^GRECC/TRACTS, VA Boston Healthcare System, Boston, MA, United States; ^33^Center for Genomics and Personalised Health, Queensland University of Technology, Kelvin Grove, QLD, Australia; ^34^Gallipoli Medical Research Foundation, Greenslopes Private Hospital, Brisbane, QLD, Australia; ^35^Department of Psychology, University of Washington, Seattle, WA, United States; ^36^School of Psychology and Counseling, Queensland University of Technology, Kelvin Grove, QLD, Australia; ^37^Department of Psychiatry and Behavioral Sciences, University of Washington, Seattle, WA, United States

**Keywords:** posttraumatic stress disorder, genetics, blood pressure, trans-ethnic, meta-analysis

## Abstract

Growing research suggests that posttraumatic stress disorder (PTSD) may be a risk factor for poor cardiovascular health, and yet our understanding of who might be at greatest risk of adverse cardiovascular outcomes after trauma is limited. In this study, we conducted the first examination of the individual and synergistic contributions of PTSD symptoms and blood pressure genetics to continuous blood pressure levels. We harnessed the power of the Psychiatric Genomics Consortium-PTSD Physical Health Working Group and investigated these associations across 11 studies of 72,224 trauma-exposed individuals of European (*n* = 70,870) and African (*n* = 1,354) ancestry. Genetic contributions to blood pressure were modeled via polygenic scores (PGS) for systolic blood pressure (SBP) and diastolic blood pressure (DBP) that were derived from a prior trans-ethnic blood pressure genome-wide association study (GWAS). Results of trans-ethnic meta-analyses revealed significant main effects of the PGS on blood pressure levels [SBP: β = 2.83, standard error (SE) = 0.06, *p* < 1E-20; DBP: β = 1.32, SE = 0.04, *p* < 1E-20]. Significant main effects of PTSD symptoms were also detected for SBP and DBP in trans-ethnic meta-analyses, though there was significant heterogeneity in these results. When including data from the largest contributing study – United Kingdom Biobank – PTSD symptoms were negatively associated with SBP levels (β = −1.46, SE = 0.44, *p* = 9.8E-4) and positively associated with DBP levels (β = 0.70, SE = 0.26, *p* = 8.1E-3). However, when excluding the United Kingdom Biobank cohort in trans-ethnic meta-analyses, there was a nominally significant positive association between PTSD symptoms and SBP levels (β = 2.81, SE = 1.13, *p* = 0.01); no significant association was observed for DBP (β = 0.43, SE = 0.78, *p* = 0.58). Blood pressure PGS did not significantly moderate the associations between PTSD symptoms and blood pressure levels in meta-analyses. Additional research is needed to better understand the extent to which PTSD is associated with high blood pressure and how genetic as well as contextual factors may play a role in influencing cardiovascular risk.

## Introduction

Cardiovascular disease (CVD) is the leading cause of death and disability worldwide (Virani et al., 2020), and longitudinal research has found that posttraumatic stress disorder (PTSD) – the quintessential trauma-related mental disorder – precedes and predicts the onset of incident CVD (Kubzansky et al., 2007, 2009; Vaccarino et al., 2013; Sumner et al., 2015). Elevated blood pressure may be one mechanism by which PTSD contributes to heightened CVD risk. Meta-analytic evidence suggests that individuals with (versus without) PTSD have significantly greater resting systolic blood pressure (SBP) and diastolic blood pressure (DBP), as well as greater DBP response to trauma-related cues (Buckley and Kaloupek, 2001; Pole, 2007). In addition, PTSD has been associated with elevated blood pressure and hypertension in a variety of trauma-exposed populations, including military veteran and community-based samples (Schnurr et al., 2000; Kibler et al., 2009; Sumner et al., 2016; Burg et al., 2017; Edmondson et al., 2018; Howard et al., 2018).

Although growing evidence suggests that PTSD may be linked to elevated blood pressure – a major risk factor for CVD – there is a lack of understanding of who may be at greatest risk. Harnessing information about genetic liability may help to shed light on who is most vulnerable to developing adverse physical health outcomes after trauma. For example, in a predominantly male sample of younger veterans, Wolf et al. (2017) found that obesity polygenic risk, reflecting additive risk for obesity based on thousands of genotypes across the genome, moderated the relation between PTSD and metabolic syndrome (an indicator of cardiometabolic risk). Specifically, lifetime PTSD symptom severity was more strongly associated with metabolic syndrome severity among individuals with greater polygenic risk for obesity (Wolf et al., 2017). Thus, examining whether genetic liability moderates associations of PTSD with physical health indicators, such as blood pressure (i.e., Gene × Environment interactions) may further our understanding of chronic disease risk and inform more targeted allocation of screening and intervention efforts.

Recent work has documented the genetic underpinnings of blood pressure in both European ancestry and trans-ethnic samples (Franceschini et al., 2013; Warren et al., 2017; Giri et al., 2019). These large-scale genome-wide association studies (GWAS) have identified over 250 genetic loci that are significantly associated with blood pressure outcomes, such as SBP and DBP. In the most recent trans-ethnic meta-analysis in over 750,000 individuals, Giri et al. (2019) replicated 216 previously identified SBP loci and 76 DBP loci, and identified 124 novel loci for SBP and 4 for DBP in analyses of common variants; a smaller number of loci were identified based on analyses of rare variants. Notably, in line with prior research (Franceschini et al., 2013), the direction of effects of these single nucleotide polymorphisms (SNPs) on blood pressure were consistent across race and ethnicity. Furthermore, polygenic scores (PGS) based on the GWAS results were significantly and positively associated with a clinical outcome – hypertension; associations were similar across racial and ethnic groups (Giri et al., 2019).

This study investigated whether blood pressure PGS moderated the associations of PTSD with SBP and DBP. Through a collaborative effort of the Psychiatric Genomics Consortium-PTSD (PGC-PTSD) Physical Health Working Group, we examined main effects of PTSD and blood pressure PGS on continuous blood pressure levels, as well as their interaction, in 72,224 trauma-exposed individuals of European and African ancestry. To estimate associations across studies and examine the role of ancestry, we conducted trans-ethnic, as well as single ancestry, meta-analyses. We hypothesized that both PTSD and blood pressure PGS would be associated with higher SBP and DBP levels and that the association between PTSD and SBP/DBP would be greatest for those with higher blood pressure PGS.

## Materials and Methods

### Cohort Descriptions

Eleven studies participating in the PGC-PTSD consortium and PGC-PTSD Physical Health Working Group contributed data to the current investigation. These studies included the CHOICE study (*N* = 78), D-cycloserine study (DCS; *N* = 80), Grady Trauma Project (GTP; *N* = 737), Gallipoli Medical Research Foundation-Queensland University of Technology (GMRF-QUT; *N* = 275), Hostility, PTSD, and Physical Health Risk Factors (HOST; *N* = 121), Marine Resiliency Study (MRS; *N* = 1,933), Optimizing Treatment for PTSD (OPT; *N* = 75), Shared Roots Study (SHRS; *N* = 292), Translational Research Center for TBI and Stress Disorders (TRACTS; *N* = 312), United Kingdom Biobank (UKBB; *N* = 67,270), and Vietnam Era Twin Study of Aging (VETSA; *N* = 1,051). Inclusion criteria were as follows: (1) study administered a measure of PTSD symptoms; (2) blood pressure was measured directly; and (3) genetic data were available. Blood pressure measurement was conducted at the same time or after PTSD assessment. If a study included more than one blood pressure measurement (i.e., measures of blood pressure at more than one time point, in contrast to multiple readings of blood pressure collected at a single time point and averaged to obtain a single blood pressure assessment), investigators selected the earliest blood pressure measure administered to participants in the study that occurred at or after the assessment of PTSD symptoms. In cohorts that received treatment (CHOICE, DCS, and OPT), PTSD and blood pressure measurements used were obtained prior to randomization. Cohort details, including information about measures of PTSD symptoms and blood pressure, are provided in the Supplementary Material.

### PTSD Symptoms

Each study administered a measure of PTSD symptoms (see Supplementary Material for details). To facilitate comparisons across studies given differences in the measures used, total PTSD symptom severity scores were rescaled to a 0–1 range based on the theoretical ranges of each inventory [(observed score – minimum possible score)/maximum possible score]. This 0–1 scale means that each individual’s score is akin to a percentage of maximum possible symptom severity and thus is directly comparable across cohorts. Examining this rescaled continuous measure of PTSD symptoms, rather than a categorical PTSD indicator, allowed us to model individual differences in severity across studies on a common scale and maximize power (e.g., MacCallum et al., 2002).

### Blood Pressure

We examined SBP and DBP (see Supplementary Material for details) as outcomes in separate analyses. As recommended by Tobin et al. (2005), antihypertensive medication use was accounted for by adding a constant to SBP and DBP values. Specifically, if use of antihypertensive medication was reported for a given participant, 15 mmHg was added to the SBP value and 10 mmHg was added to the DBP value, as in prior research (Tobin et al., 2005; Warren et al., 2017). CHOICE, DCS, and UKBB did not include a measure of antihypertensive medication, and thus blood pressure values were not adjusted in these cohorts.

### Genotype Quality Control, Imputation, and Ancestry Determination

Quality control for genotypes was conducted following standard procedures and imputed to the 1,000 Genomes Phase 3 reference panel, as previously described in detail (Bycroft et al., 2018; Nievergelt et al., 2019).

Global ancestry was determined using the program SNPweights (Chen et al., 2013), using a reference panel of 10,000 ancestry informative markers measured in 71 reference populations and 6 continental groups, as previously described (Nievergelt et al., 2019). Pre-quality control genotypes were used for these analyses. The current study focused on participants of European and African ancestry. European ancestry participants were defined as those with >90% European ancestry and <5% ancestry in the remaining reference populations. African American participants were defined as those with >10% African ancestry and <5% ancestry in the other non-European ancestry populations (i.e., placing them on an African-European population gradient), and they were analyzed in the African ancestry group. Participants in SHRS were classified broadly as “South African” and included in the African ancestry group; strong outliers [± 4 standard deviations (SDs) of the mean principal component (PC) for any of the first five ancestry PCs] were removed.

### Blood Pressure PGS

Blood pressure PGS were calculated using PRSice v2.3.3 (Choi and O’Reilly, 2019) based on effect sizes from the trans-ethnic GWAS of independent samples from the Million Veteran Program (Giri et al., 2019). SBP and DBP GWAS summary statistics were used to calculate SBP and DBP PGS, respectively^1^. We filtered the summary statistics to remove SNPs with minor allele frequency (MAF) < 1%, SNPs with effects estimated in less than 90% of the total sample size, or strand ambiguous SNPs. Imputed genotypes for PGC-PTSD datasets were converted from probabilities to hard calls, where the genotype called was the one with the highest imputation probability. If the highest imputation probability was < 0.8, the genotype call was instead set to missing. Only markers with < 5% missing rate and MAF > 1% (calculated within each dataset) were retained. For computation of PGS, variants were linkage disequilibrium (LD) clumped (LD information was calculated within each dataset) based on a 250 kb window where only variants with *r*^2^ < 0.1 were retained. PGS were calculated at multiple *p*-value thresholds (*p*T), varying by increments of 0.01 and ranging from 5E-8 to 1.

### Covariates

Each study provided information on participant age (in years) and biological sex. In addition, ancestry PCs were calculated. Specifically, within each dataset, related participants were identified using identity by state estimation in PLINK 1.9 (Purcell et al., 2007). For every related pair (pi_hat > 0.2), one individual was removed. PCs were calculated in unrelated participants of the same genetically determined global ancestry using the smartPCA algorithm implemented in Eigenstrat 6.1.4 (Price et al., 2006). For PC calculation, SNPs were excluded if they had MAF < 5%, Hardy Weinberg Equilibrium *p* > 1E-3, or ambiguous strand, or were located in the major histocompatibility complex region (chr. 6, 25–35 MB) or chromosome 8 inversion (chr. 8, 7–13 MB).

### Analytic Approach

To enhance comparability of the blood pressure PGS used across cohorts, we identified the best fitting PGS *p*T for the largest sample: UKBB. In this sample, the various PGS were tested for associations with their corresponding blood pressure measure in linear models adjusting for the first five ancestry PCs, age, age-squared, and sex. The best-fitting thresholds in UKBB were then used for all analyses of blood pressure PGS in the remaining datasets. To make effect estimates comparable, PGS were centered to the UKBB cohort sample means and SDs.

Analyses were performed stratified by cohort. As in prior research in the PGC-PTSD (Nievergelt et al., 2019), CHOICE, DCS, and OPT – three treatment studies that contributed pre-treatment assessments of PTSD and blood pressure for the current study – were analyzed together as a single cohort. Main effects of (1) blood pressure PGS and (2) PTSD symptoms were each tested using linear regression models with blood pressure levels as the outcome, adjusting for the first five ancestry PCs, age, age-squared, and sex (single-sex studies did not include a sex covariate). As the VETSA study contained twin pairs, data were analyzed using a linear mixed model accounting for non-independence of observations, as implemented in the lme4 R package (Bates et al., 2015). Interactions of PTSD symptoms and blood pressure PGS were tested by fitting a linear model with main effects of PTSD symptoms, blood pressure PGS, and the interaction term all included in the model. Interactions of all covariates with PTSD symptoms and with blood pressure PGS were included as covariates for these models per the recommendation of Keller (2014), along with covariate main effects^2^.

Effect estimates from each cohort were meta-analyzed using fixed effects inverse variance weighted meta-analysis, implemented in the metafor R package (Viechtbauer, 2010). We conducted trans-ethnic meta-analyses including all cohorts, in addition to separate meta-analyses of the African ancestry and European ancestry participants. To assess results for heterogeneity, Cochran’s *Q* and the *I*^2^ statistic were calculated (Higgins et al., 2003). Cochran’s *Q* statistic tests the null hypothesis that the included studies are evaluating the same effect; a *p*-value is generated by comparing the *Q* statistic with a χ^2^ distribution with degrees of freedom equal to the number of studies minus 1. However, the *Q* statistic is influenced by the number of studies included in a meta-analysis (Higgins et al., 2003). As a result, the *I*^2^ statistic, which quantifies the percent of total variability across studies that is due to heterogeneity rather than chance, is frequently also calculated when assessing heterogeneity. The *I*^2^ statistic ranges from 0 to 100%, with values of 25, 50, and 75% suggesting low, moderate, and high heterogeneity, respectively (Higgins et al., 2003). Diagnostic plots were produced and assessed for fit and influence of potential outliers. As the UKBB sample alone was substantially larger than the combined remaining cohorts, meta-analyses were performed with and without the UKBB sample, and *z*-tests were used to compare effect size estimate differences between meta-analyses including and excluding the UKBB sample.

We also conducted several secondary analyses to further explore our results. First, sex-specific PGS and PTSD effects were tested in UKBB (i.e., the largest cohort) by conducting sex-stratified analyses and by fitting Sex × PTSD Symptoms and Sex × Blood Pressure PGS interaction terms in models with PTSD symptoms and blood pressure PGS main effects in the full cohort. Again, per Keller (2014), interactions of all covariates with the variables in interactions were included in models testing interactions. Second, we conducted meta-analyses stratified by cohort type – military or community-based – to investigate whether the nature of the sample influenced results. For these analyses, we excluded the HOST study and combined CHOICE, DCS, and OPT cohort, as they enrolled both military and civilian participants (included cohorts are described in Supplementary Table 2). Third, as some studies (UKBB, CHOICE, and DCS) did not assess antihypertensive medication use and thus contributed unadjusted blood pressure values to meta-analyses, we restricted meta-analyses to those studies with adjusted blood pressure values based on the presence of antihypertensive medication use. With these analyses, we were able to examine the patterns of results that emerged when recommended guidelines for accounting for antihypertensive medication use (Tobin et al., 2005) were followed across all studies.

## Results

### Cohort Characteristics

Demographic information, PTSD symptom severity, and blood pressure levels for each study, stratified by ancestry, are presented in Table 1. There was a mix of military and community-based cohorts, and several of the military cohorts were all-male (e.g., GMRF-QUT, MRS, and VETSA). Although individuals of European and African ancestry were included in these analyses, the overall sample was predominantly of European ancestry due to the large sample size of UKBB. On average, participants were in young and middle adulthood, although GMRF-QUT, UKBB, and VETSA had older average ages. Average PTSD symptoms on the transformed 0–1 scale varied across studies, with the lowest mean symptom levels detected for GMRF-QUT and UKBB and the highest mean symptom levels detected for TRACTS and the three treatment studies: CHOICE, DCS, and OPT. Across studies, mean transformed PTSD symptom levels were higher for African ancestry participants than for European ancestry participants as well. For studies that included both European and African ancestry participants (CHOICE, DCS, HOST, MRS, and OPT), we computed the difference in the mean transformed PTSD symptoms for individuals of African and European ancestry. In a meta-analysis of these difference scores, there was a statistically significant difference such that individuals of African ancestry had higher PTSD symptom levels than individuals of European ancestry (difference = 0.02, SE = 0.00, *z* = 4.10, *p* < 1E-4). For the majority of studies, mean SBP and DBP were in the normotensive range. Average blood pressure levels were similar for participants of European and African ancestry. For those studies reporting antihypertensive medication use, prevalence in cohorts ranged from 0% in MRS to 57% in VETSA.

**TABLE 1:** Participant characteristics for each contributing study, stratified by ancestry (*N* = 72,224).

Study	Sample size	Age	Male sex	Cohort type	PTSD measure	PTSD symptoms raw		PTSD symptoms rescaled^*a*^	SBP	DBP	Antihypertensive medication use
										
		M (SD)	*n* (%)			M (SD)	Range^*b*^	M (SD)	M (SD)	M (SD)	*n* (%)
**African ancestry**											
CHOICE	28	39.9 (9.9)	6 (21)	Community	PSS-I	30.54 (5.92)	18–39	0.60 (0.12)	120.18 (16.07)	78.39 (10.14)	N/A
DCS	36	36.2 (8.5)	33 (92)	Military	PSS-SR	39.00 (17.83)	19–50	0.76 (0.35)	127.14 (15.51)	86.00 (12.12)	N/A
GTP	737	42.5 (12.1)	210 (29)	Community	mPSS	16.80 (14.22)	0–51	0.33 (0.28)	129.14 (19.87)	76.79 (12.06)	275 (37)
HOST	71	30.1 (5.8)	32 (45)	Military and community	CAPS	41.11 (27.20)	0–109	0.38 (0.25)	129.15 (12.64)	77.45 (9.01)	3 (4)
MRS	152	24.1 (4.0)	152 (100)	Military	CAPS	21.18 (18.48)	0–95	0.16 (0.14)	125.79 (11.18)	72.53 (8.33)	0 (0)
OPT	38	38.3 (11.7)	10 (26)	Community	PSS-I	33.92 (4.80)	25–43	0.67 (0.09)	130.47 (18.22)	87.27 (12.28)	4 (11)
SHRS	292	41.8 (12.1)	75 (26)	Community	PCL-5	32.83 (24.04)	0–79	0.44 (0.30)	126.21 (17.77)	81.03 (11.78)	58 (20)
Sub-group *n*	1,354										
**European ancestry**											
CHOICE	50	38.0 (12.5)	9 (18)	Community	PSS-I	30.62 (6.38)	17–45	0.61 (0.60)	122.00 (17.42)	78.16 (9.15)	N/A
DCS	44	32.6 (6.6)	42 (95)	Military	PSS-SR	33.42 (11.13)	10–38	0.66 (0.22)	124.84 (12.47)	87.00 (10.94)	N/A
GMRF-QUT	275	68.7 (3.9)	275 (100)	Military	CAPS-5	9.74 (10.10)	0–56	0.07 (0.07)	141.67 (17.18)	79.92 (8.28)	37 (13)
HOST	50	29.3 (5.2)	29 (58)	Military and Community	CAPS	41.27 (30.99)	0–112	0.37 (0.28)	120.27 (13.96)	72.01 (7.35)	3 (6)
MRS	1,781	23.4 (3.3)	1,781 (100)	Military	CAPS	19.10 (18.52)	0–117	0.14 (0.14)	123.36 (11.71)	71.00 (9.80)	0 (0)
OPT	37	36.0 (11.8)	7 (19)	Community	PSS-I	32.49 (4.74)	23–42	0.64 (0.09)	127.27 (18.81)	84.32 (13.81)	4 (11)
TRACTS	312	31.98 (8.4)	292 (94)	Military	CAPS	69.44 (33.10)	0–115	0.53 (0.25)	118.02 (11.89)	76.04 (9.63)	14 (4)
UKBB	67,270	55.8 (7.7)	31,210 (46)	Community	PCL-6	8.41 (3.44)	6–30	0.10 (0.14)	137.62 (18.96)	81.58 (10.54)	N/A
VETSA	1,051	61.8 (2.4)	1,051 (100)	Military	PCL	26.02 (10.36)	17–84	0.13 (0.15)	128.10 (15.00)	77.57 (8.42)	596 (57)
Sub-group *n*	70,870										

*CAPS = Clinician-Administered PTSD Scale for DSM-IV; CAPS-5 = Clinician-Administered PTSD Scale for DSM-5; DCS = D-cycloserine Study; DBP = diastolic blood pressure; GMRF-QUT = Gallipoli Medical Research Foundation-Queensland University of Technology; GTP = Grady Trauma Project; HOST = Hostility, PTSD, and Physical Health Risk Factors; M = mean; mPSS = Modified PTSD Symptom Scale; MRS = Marine Resiliency Study; N/A = not applicable; OPT = Optimizing Treatment for PTSD; PCL = PTSD Checklist; PCL-5 = PTSD Checklist for DSM-5; PCL-6 = PTSD Checklist-6 item version; PSS-I = PTSD Symptom Scale-Interview; PSS-SR = PTSD Symptom Scale, Self-report version; PTSD = posttraumatic stress disorder; SBP = systolic blood pressure; SD = standard deviation; SHRS = Shared Roots Study; TRACTS = Translational Research Center for TBI and Stress Disorders; UKBB = United Kingdom Biobank; VETSA = Vietnam Era Twin Study of Aging. ^*a*^The rescaled PTSD symptom severity score ranged from 0 to 1. ^*b*^Range is the observed range of PTSD symptoms in the cohort.*

### Main Effects of Blood Pressure PGS

Polygenic scores for SBP and DBP were calculated using the Million Veteran Program blood pressure GWAS results (Giri et al., 2019) and were used to predict SBP and DBP, respectively, in the different cohorts. As noted above, the optimal *p*Ts were selected based on results from the largest study: UKBB. The optimal *p*T for predicting SBP in UKBB was *p* ≤ 0.02, where PGS explained 2.56% of the variation in SBP (*p* < 1E-100). Every 1 SD increase of PGS relative to the sample mean was associated with a 3.03-point increase in SBP (SE = 0.07, *p* < 1E-20). The optimal *p*T for predicting DBP in UKBB was *p* ≤ 0.01; PGS explained 1.66% of the variation in DBP (*p* < 1E-100). For every 1 SD increase of PGS relative to the sample mean, there was a 1.37-point increase in DBP (SE = 0.04, *p* < 1E-20).

**FIGURE 1:**
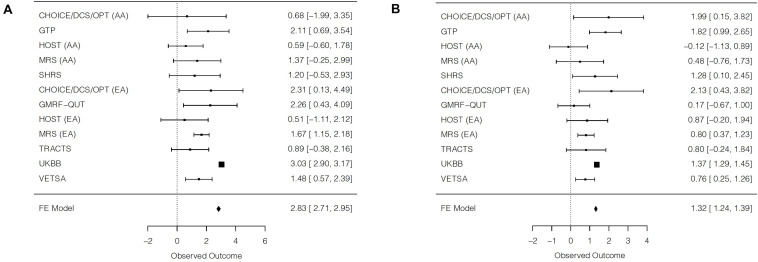
Forest plots of trans-ethnic meta-analyses for the main effect of blood pressure polygenic scores on **(A)** systolic blood pressure and **(B)** diastolic blood pressure. For each cohort, a square is plotted at the effect estimate value. The size of each plotted square reflects relative precision, where size is inversely proportional to the standard error of a given cohort. Reported effects are beta coefficients and 95% confidence intervals. AA = African ancestry; EA = European ancestry; FE = fixed effects.

In trans-ethnic meta-analyses, the PGS significantly predicted blood pressure (SBP: β = 2.83, SE = 0.06, *p* < 1E-20; DBP: β = 1.32, SE = 0.04, *p* < 1E-20; Figures 1A,B), although there was substantial heterogeneity (Table 2). In the trans-ethnic meta-analyses, effect sizes of the PGS on blood pressure were significantly smaller, albeit still significant and positively related to blood pressure, when UKBB was not included in analyses (SBP: *z* = −8.17, *p* < 3.8E-16; DBP: *z* = −4.27, *p* < 2.0E-5). Trans-ethnic meta-analyses of studies excluding UKBB indicated a 1.46-point increase in SBP for every 1 SD increase in PGS (SE = 0.18, *p* = 8.8E-17) and a 0.83-point increase in DBP for every 1 SD increase in PGS (SE = 0.12, *p* = 2.1E-11; Supplementary Figures 1A,B). No statistically significant heterogeneity was detected in the trans-ethnic meta-analyses of cohorts without UKBB, although there was evidence of low-to-moderate heterogeneity that persisted for DBP. Results of separate meta-analyses of African ancestry and European ancestry participants were similar, overall, to the trans-ethnic meta-analyses without UKBB, although moderate-to-high heterogeneity was observed in the African ancestry meta-analysis for DBP.

**TABLE 2:** Main effects of polygenic scores on blood pressure, within and across cohorts.

Individual cohort results	β (SE)	*t*	*p*			
**Systolic blood pressure**						
CHOICE/DCS/OPT AA	0.68 (1.36)	0.50	0.62			
GTP AA	2.11 (0.73)	2.91	3.8E-3			
HOST AA	0.59 (0.61)	0.97	0.34			
MRS AA	1.37 (0.83)	1.66	0.10			
SHRS AA	1.20 (0.88)	1.36	0.18			
CHOICE/DCS/OPT EA	2.31 (1.11)	2.07	0.04			
GMRF-QUT EA	2.26 (0.93)	2.43	0.02			
HOST EA	0.51 (0.82)	0.62	0.54			
MRS EA	1.67 (0.26)	6.30	3.8E-10			
TRACTS EA	0.89 (0.65)	1.37	0.17			
UKBB EA	3.03 (0.07)	45.2	<1E-20			
VETSA EA	1.48 (0.46)	3.20	1.4E-3			

**Meta-analysis results**	**β (SE)**	***z***	***p***	***Q***	***p*-het**	***I* ^2^**

Trans-ethnic meta-analysis	2.83 (0.06)	45.16	<1E-20	78.22	3.3E-12	85.9
Trans-ethnic meta-analysis without UKBB	1.46 (0.18)	8.32	8.8E-17	7.35	0.69	0
AA meta-analysis	1.20 (0.36)	3.38	7.2E-4	2.79	0.59	0
EA meta-analysis without UKBB	1.54 (0.20)	7.64	2.2E-14	3.89	0.56	0

**Individual cohort results**	**β (SE)**	***t***	***p***			

**Diastolic blood pressure**						
CHOICE/DCS/OPT AA	1.99 (0.94)	2.12	0.04			
GTP AA	1.82 (0.42)	4.28	2.1E-5			
HOST AA	−0.12 (0.51)	−0.24	0.81			
MRS AA	0.48 (0.63)	0.77	0.44			
SHRS AA	1.28 (0.60)	2.13	0.03			
CHOICE/DCS/OPT EA	2.13 (0.86)	2.46	0.02			
GMRF-QUT EA	0.17 (0.43)	0.39	0.69			
HOST EA	0.87 (0.55)	1.59	0.12			
MRS EA	0.80 (0.22)	3.63	2.9E-4			
TRACTS EA	0.80 (0.53)	1.52	0.13			
UKBB EA	1.37 (0.04)	34.40	<1E-20			
VETSA EA	0.76 (0.26)	2.94	3.3E-3			

**Meta-analysis results**	**β (SE)**	***z***	***p***	***Q***	***p*-het**	***I* ^2^**

Trans-ethnic meta-analysis	1.32 (0.04)	34.84	<1E-20	33.08	5.0E-4	66.8
Trans-ethnic meta-analysis without UKBB	0.83 (0.12)	6.70	2.1E-11	15.99	0.10	37.5
AA meta-analysis	1.06 (0.25)	4.20	2.7E-5	10.40	0.03	61.6
EA meta-analysis without UKBB	0.76 (0.14)	5.33	9.8E-8	4.51	0.48	0

*Models adjusted for the first five ancestry principal components, age, age-squared, and sex (for cohorts with men and women). AA = African ancestry; DCS = D-cycloserine Study; EA = European ancestry; GMRF-QUT = Gallipoli Medical Research Foundation-Queensland University of Technology; GTP = Grady Trauma Project; HOST = Hostility, PTSD, and Physical Health Risk Factors; MRS = Marine Resiliency Study; OPT = Optimizing Treatment for PTSD; *p*-het = *p*-value for Cochran’s *Q*; *Q* = Cochran’s *Q*; SE = standard error; SHRS = Shared Roots Study; TRACTS = Translational Research Center for TBI and Stress Disorders; UKBB = UK Biobank; VETSA = Vietnam Era Twin Study of Aging.*

### Main Effects of PTSD Symptoms

**FIGURE 2:**
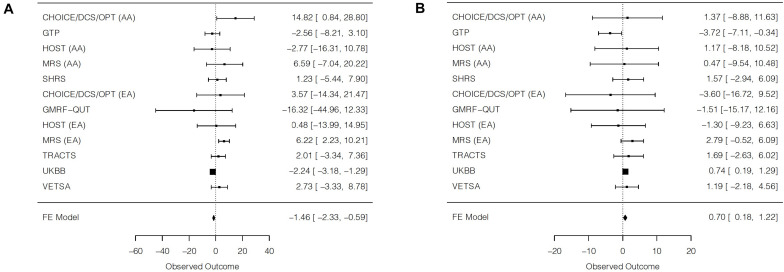
Forest plots of trans-ethnic meta-analyses for the main effect of posttraumatic stress disorder symptoms on **(A)** systolic blood pressure and **(B)** diastolic blood pressure. For each cohort, a square is plotted at the effect estimate value. The size of each plotted square reflects relative precision, where size is inversely proportional to the standard error of a given cohort. Reported effects are beta coefficients and 95% confidence intervals. AA = African ancestry; EA = European ancestry; FE = fixed effects.

Significant main effects of PTSD symptoms on SBP were detected in three individual cohorts, although the effect directions differed (Table 3). Whereas PTSD symptoms were negatively associated with SBP levels in UKBB, PTSD symptoms were positively associated with SBP levels in European ancestry participants from MRS and in African ancestry participants from the CHOICE/DCS/OPT combined cohort. The trans-ethnic meta-analysis of all cohorts was statistically significant and indicated a negative association between PTSD symptoms and SBP (β = −1.46, SE = 0.44, *p* = 9.8E-4; Figure 2A). An individual with the highest possible PTSD symptom severity was predicted to have SBP that was 1.46 mmHg lower than an individual with the lowest possible PTSD symptom severity. However, this finding was largely driven by the UKBB results, and there was substantial heterogeneity detected. In the trans-ethnic meta-analysis of all cohorts excluding UKBB, there was a nominally significant positive association between PTSD symptoms and SBP (β = 2.81, SE = 1.13, *p* = 0.01; Supplementary Figure 2A). The effect sizes for the main effect of PTSD symptoms on SBP levels differed significantly when including vs. excluding UKBB (*z* = −4.11, *p* = 4.0E-5). Further, when excluding UKBB in trans-ethnic meta-analyses, heterogeneity was minimal and not statistically significant. A significant positive main effect of PTSD symptoms on SBP was also detected in the European ancestry meta-analysis without UKBB (β = 3.94, SE = 1.39, *p* = 4.7E-3). No significant main effect of PTSD symptoms was detected for SBP for the African ancestry meta-analysis (β = 0.63, SE = 1.93, *p* = 0.74).

**TABLE 3:** Main effects of PTSD symptoms on blood pressure, within and across cohorts.

Individual cohort results	β (SE)	*t*	*p*			
**Systolic blood pressure**						
CHOICE/DCS/OPT AA	14.82 (7.13)	2.08	0.04			
GTP AA	−2.56 (2.89)	−0.89	0.38			
HOST AA	−2.77 (6.91)	−0.40	0.69			
MRS AA	6.59 (6.95)	0.95	0.35			
SHRS AA	1.23 (3.40)	0.36	0.72			
CHOICE/DCS/OPT EA	3.57 (9.14)	0.39	0.70			
GMRF-QUT EA	−16.32 (14.62)	−1.12	0.27			
HOST EA	0.48 (7.38)	0.06	0.95			
MRS EA	6.22 (2.04)	3.05	2.3E-3			
TRACTS EA	2.01 (2.73)	0.74	0.46			
UKBB EA	−2.24 (0.48)	−4.64	3.4E-6			
VETSA EA	2.73 (3.09)	0.88	0.38			

**Meta-analysis results**	**β (SE)**	***z***	***p***	***Q***	***p*-het**	***I* ^2^**

Trans-ethnic meta-analysis	−1.46 (0.44)	−3.30	9.8E-4	29.03	2.0E-3	62.1
Trans-ethnic meta-analysis without UKBB	2.81 (1.13)	2.49	0.01	12.16	0.27	17.8
AA meta-analysis	0.63 (1.93)	0.33	0.74	6.18	0.19	35.3
EA meta-analysis without UKBB	3.94 (1.39)	2.83	4.7E-3	4.05	0.54	0

**Individual cohort results**	**β (SE)**	***t***	***p*-value**			

**Diastolic blood pressure**						
CHOICE/DCS/OPT AA	1.37 (5.23)	0.26	0.79			
GTP AA	−3.72 (1.73)	−2.16	0.03			
HOST AA	1.17 (4.77)	0.25	0.81			
MRS AA	0.47 (5.11)	0.09	0.93			
SHRS AA	1.57 (2.30)	0.68	0.50			
CHOICE/DCS/OPT EA	−3.60 (6.69)	−0.54	0.59			
GMRF-QUT EA	−1.51 (6.97)	−0.22	0.83			
HOST EA	−1.30 (4.05)	−0.32	0.75			
MRS EA	2.79 (1.69)	1.65	0.10			
TRACTS EA	1.69 (2.21)	0.77	0.44			
UKBB EA	0.74 (0.28)	2.62	0.01			
VETSA EA	1.19 (1.72)	0.69	0.38			

**Meta-analysis results**	**β (SE)**	***z***	***p*-value**	***Q***	***p*-het**	***I* ^2^**

Trans-ethnic meta-analysis	0.70 (0.26)	2.65	8.1E-3	9.34	0.59	0
Trans-ethnic meta-analysis without UKBB	0.43 (0.78)	0.55	0.58	9.20	0.51	0
AA meta-analysis	−1.30 (1.25)	−1.04	0.30	4.18	0.38	4.3
EA meta-analysis without UKBB	1.54 (1.00)	1.54	0.12	1.87	0.87	0

*Models adjusted for the first five ancestry principal components, age, age-squared, and sex (for cohorts with men and women). AA = African ancestry; DCS = D-cycloserine Study; EA = European ancestry; GMRF-QUT = Gallipoli Medical Research Foundation-Queensland University of Technology; GTP = Grady Trauma Project; HOST = Hostility, PTSD, and Physical Health Risk Factors; MRS = Marine Resiliency Study; OPT = Optimizing Treatment for PTSD; *p*-het = *p*-value for Cochran’s *Q*; PTSD = posttraumatic stress disorder; *Q* = Cochran’s *Q*; SE = standard error; SHRS = Shared Roots Study; TRACTS = Translational Research Center for TBI and Stress Disorders; UKBB = United Kingdom Biobank; VETSA = Vietnam Era Twin Study of Aging.*

For DBP, there were significant main effects of PTSD symptoms in the UKBB and GTP cohorts, but effects were in the opposite direction. In UKBB, PTSD symptoms were positively associated with DBP levels, but PTSD symptoms were negatively associated with DBP levels in GTP. The trans-ethnic meta-analysis was statistically significant and suggested a small positive association between PTSD symptoms and DBP (β = 0.70, SE = 0.26, *p* = 8.1E-3; Figure 2B). An individual with the highest possible PTSD symptom severity was predicted to have a DBP measure that was 0.70 mmHg higher than an individual with the lowest possible PTSD symptom severity. No statistically significant heterogeneity was detected. In the trans-ethnic meta-analysis excluding UKBB, the association remained positive but was smaller and not statistically significant (β = 0.43, SE = 0.78, *p* = 0.58; Supplementary Figure 2B). However, the effect sizes for the main effect of PTSD symptoms on DBP levels did not differ significantly when including vs. excluding UKBB (*z* = 0.37, *p* = 0.71). A larger positive, albeit non-significant, main effect of PTSD symptoms was observed in the European ancestry meta-analysis without UKBB (β = 1.54, SE = 1.00, *p* = 0.12), whereas a negative, non-significant main effect of PTSD symptoms was observed in the African ancestry meta-analysis (β = −1.30, SE = 1.25, *p* = 0.30).

### Interactions of PTSD Symptoms and Blood Pressure PGS

**FIGURE 3:**
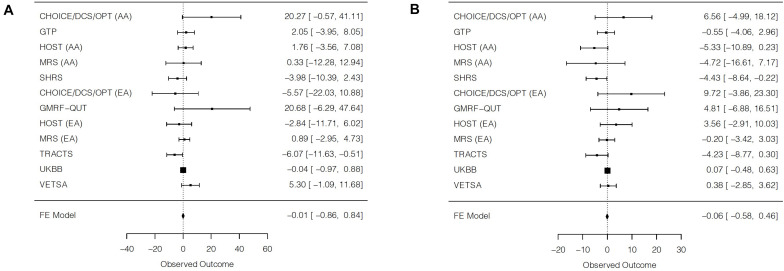
Forest plots of trans-ethnic meta-analyses for the interaction of posttraumatic stress disorder symptoms and blood pressure polygenic scores on **(A)** systolic blood pressure and **(B)** diastolic blood pressure. For each cohort, a square is plotted at the effect estimate value. The size of each plotted square reflects relative precision, where size is inversely proportional to the standard error of a given cohort. Reported effects are beta coefficients and 95% confidence intervals. AA = African ancestry; EA = European ancestry; FE = fixed effects.

Despite nominally significant interaction terms of PTSD symptoms and blood pressure PGS for TRACTS for SBP and for SHRS for DBP, no significant interaction effects emerged in trans-ethnic meta-analyses (Table 4 and Figure 3). Furthermore, there was evidence of low-to-moderate heterogeneity that did not reach the level of statistical significance. For DBP, there was a nominally significant interaction of PTSD symptoms with PGS for the African ancestry meta-analysis (β = −2.44, SE = 1.19, *p* = 0.04).

**TABLE 4:** Interaction effects of PTSD symptoms with polygenic scores on blood pressure, within and across cohorts.

Individual cohort results	β (SE)	*t*	*p*-value			
**Systolic blood pressure**						
CHOICE/DCS/OPT AA	20.27 (10.63)	1.91	0.06			
GTP AA	2.05 (3.06)	0.67	0.50			
HOST AA	1.76 (2.72)	0.65	0.52			
MRS AA	0.33 (6.44)	0.05	0.96			
SHRS AA	−3.98 (3.27)	−1.22	0.22			
CHOICE/DCS/OPT EA	−5.57 (8.39)	−0.66	0.51			
GMRF-QUT EA	20.68 (13.76)	1.50	0.13			
HOST EA	−2.84 (4.52)	−0.63	0.53			
MRS EA	0.89 (1.96)	0.45	0.65			
TRACTS EA	−6.07 (2.84)	−2.14	0.03			
UKBB EA	−0.04 (0.47)	−0.09	0.93			
VETSA EA	5.30 (3.26)	1.63	0.10			

**Meta-analysis results**	β **(SE)**	***Z***	***p*-value**	***Q***	***p*-het**	***I* ^2^**

Trans-ethnic meta-analysis	−0.01 (0.43)	−0.02	0.98	16.52	0.12	33.4
Trans-ethnic meta-analysis without UKBB	0.15 (1.04)	0.15	0.88	16.49	0.09	39.4
AA meta-analysis	0.74 (1.65)	0.45	0.65	5.78	0.22	30.8
EA meta-analysis without UKBB	−0.24 (1.35)	−0.18	0.86	10.50	0.06	52.4

**Individual cohort results**	β **(SE)**	***t***	***p*-value**			

**Diastolic blood pressure**						
CHOICE/DCS/OPT AA	6.56 (5.90)	1.11	0.27			
GTP AA	−0.55 (1.79)	−0.31	0.76			
HOST AA	−5.33 (2.84)	−1.88	0.07			
MRS AA	−4.72 (6.07)	−0.78	0.44			
SHRS AA	−4.43 (2.15)	−2.06	0.04			
CHOICE/DCS/OPT EA	9.72 (6.93)	1.40	0.16			
GMRF-QUT EA	4.81 (5.97)	0.81	0.42			
HOST EA	3.56 (3.30)	1.08	0.29			
MRS EA	−0.20 (1.65)	−0.12	0.90			
TRACTS EA	−4.23 (2.31)	−1.83	0.07			
UKBB EA	0.07 (0.28)	0.26	0.79			
VETSA EA	0.38 (1.65)	0.23	0.82			

**Meta-analysis results**	β **(SE)**	***Z***	***p*-value**	***Q***	***p*-het**	***I* ^2^**

Trans-ethnic meta-analysis	−0.06 (0.26)	−0.23	0.82	16.94	0.11	35.1
Trans-ethnic meta-analysis without UKBB	−1.01 (0.75)	−1.35	0.18	15.11	0.13	33.8
AA meta-analysis	−2.44 (1.19)	−2.06	0.04	5.48	0.24	27.0
EA meta-analysis without UKBB	−0.06 (0.97)	−0.06	0.95	7.20	0.21	30.6

*Models adjusted for the first five ancestry principal components, age, age-squared, sex (for cohorts with men and women), and interactions of all covariates with PTSD symptoms and with polygenic scores. AA = African ancestry; DCS = D-cycloserine Study; EA = European ancestry; GMRF-QUT = Gallipoli Medical Research Foundation-Queensland University of Technology; GTP = Grady Trauma Project; HOST = Hostility, PTSD, and Physical Health Risk Factors; MRS = Marine Resiliency Study; OPT = Optimizing Treatment for PTSD; *p*-het = *p*-value for Cochran’s *Q*; PTSD = posttraumatic stress disorder; *Q* = Cochran’s *Q*; SE = standard error; SHRS = Shared Roots Study; TRACTS = Translational Research Center for TBI and Stress Disorders; UKBB = United Kingdom Biobank; VETSA = Vietnam Era Twin Study of Aging.*

### Secondary Analyses

In the largest study examined – UKBB – we were able to conduct sex-stratified analyses in order to explore potential sex-specific effects (Supplementary Table 1). Women had a stronger positive association of PGS with SBP levels than men, and there was a significant Sex × PGS interaction (β = −0.42, SE = 0.14, *p* = 2.0E-3). A similar finding was observed for DBP, although the Sex × PGS interaction was only nominally significant (β = −0.20, SE = 0.08, *p* = 0.01). The negative association between PTSD symptoms and SBP levels was somewhat larger for men than for women, although there was no significant Sex × PTSD Symptoms interaction (β = 1.27, SE = 0.98, *p* = 0.19). Furthermore, even though PTSD symptoms were significantly positively associated with DBP levels in women only, the Sex × PTSD Symptoms interaction was not significant (β = 0.24, SE = 0.57, *p* = 0.68). Similar to the overall trans-ethnic meta-analyses, no significant PTSD Symptom × PGS interactions emerged in sex-stratified analyses in UKBB.

The patterns of results from meta-analyses stratified by military or community-based cohort type paralleled those of the trans-ethnic meta-analyses when excluding versus including UKBB, respectively (Supplementary Table 2). Both sets of analyses identified significant positive associations of blood pressure PGS with SBP and DBP, although effects were larger in the community-based cohorts. With respect to the main effect of PTSD symptoms, there was a significant negative effect of PTSD on SBP in the community-based cohorts (β = −2.18, SE = 0.47, *p* = 3.7E-6) and a significant positive effect of PTSD on SBP in the military cohorts (β = 4.18, SE = 1.41, *p* = 2.9E-3). For DBP, the positive main effect of PTSD was nominally significant for community-based cohorts (β = 0.64, SE = 0.28, *p* = 0.02) but not for military cohorts (β = 1.80, SE = 1.02, *p* = 0.08). No significant PTSD Symptoms × PGS interactions emerged in analyses stratified by military or community-based cohort type.

When restricting analyses to studies that assessed for antihypertensive medication use and were therefore able to adjust blood pressure values for medication use, results of trans-ethnic and single ancestry meta-analyses indicated significant positive main effects of PGS on SBP and DBP (Supplementary Table 3). Additionally, there were significant positive main effects of PTSD on SBP for the trans-ethnic and European ancestry meta-analyses, although no significant effects of PTSD were observed for DBP. No significant PTSD Symptoms × PGS interactions were detected in any of these secondary meta-analyses.

## Discussion

A substantial body of research suggests that PTSD may be a risk factor for poor cardiovascular health (Edmondson and von Känel, 2017; Koenen et al., 2017), and yet our understanding of who might be at greatest risk of adverse cardiovascular outcomes after trauma is limited. In the current study, we conducted the first examination of the individual and synergistic contributions of PTSD symptoms and genetics to continuous blood pressure levels. To explore this research question, we harnessed the power of the collaborative, consortium-based PGC-PTSD Physical Health Working Group and investigated these associations across 11 studies of over 72,000 individuals of European and African ancestry. In trans-ethnic meta-analyses, we identified polygenic main effects based on the results of a trans-ethnic blood pressure GWAS on both SBP and DBP, with higher PGS predicting higher blood pressure levels. Significant main effects of PTSD symptoms were also detected for SBP and DBP in meta-analyses, though there was substantial heterogeneity in the results across cohorts. When including data from the largest contributing study – UKBB – PTSD symptoms were negatively associated with SBP levels and positively associated with DBP levels. However, when excluding UKBB, there was a nominally significant positive association of PTSD symptoms with SBP levels; no significant association was observed for DBP. Furthermore, we did not find evidence that blood pressure PGS significantly moderated the associations between PTSD symptoms and blood pressure levels in meta-analyses.

A notable feature of our study is that we conducted trans-ethnic meta-analyses and used the results from a trans-ethnic blood pressure GWAS from the Million Veteran Program (Giri et al., 2019) to derive our blood pressure PGS. Indeed, compared to other genomics consortia, the PGC-PTSD Group is unique in that participants of diverse ancestries comprise a substantial proportion of the sample (Nievergelt et al., 2019). This aspect of our study thus enhances the generalizability of our findings and is notable given the relative lack of racial and ethnic diversity that is often observed in genetics studies (Popejoy and Fullerton, 2016), which can contribute to health disparities in underrepresented groups. As expected, higher SBP and DBP PGS were each associated with higher SBP and DBP levels, respectively. In their GWAS results, Giri et al. (2019) found that significant sentinel SNPs at independent loci accounted for more variance in SBP (3.56%) than for DBP (1.06%), and we found that effect sizes of PGS on blood pressure levels – although small – were larger for SBP than for DBP in trans-ethnic meta-analyses. However, we found that the PGS based on the best fitting *p*-value thresholds in UKBB accounted for slightly less variance in SBP and slightly more variance in DBP in this cohort compared to that observed for the sentinel SNPs in the Giri et al. (2019) GWAS. When making these comparisons, it is worth noting though that our approach of generating PGS across thousands of SNPs based on GWAS summary statistics from an independent sample differed from the approach of Giri et al. (i.e., examining variance accounted for by significant sentinel SNPs identified in their own samples). In single ancestry meta-analyses, associations were observed in both European and African ancestry individuals. These findings parallel research on the genetic underpinnings of blood pressure in individuals of diverse backgrounds, which has found similar directions of effects across a number of ancestry groups (Franceschini et al., 2013; Giri et al., 2019). We also found some evidence that PGS for SBP and, to a lesser extent, DBP were more strongly related to the corresponding blood pressure levels in women than in men in the UKBB cohort. Given sex differences in CVD (Virani et al., 2020), such findings are intriguing and merit replication and further investigation.

Although PTSD symptoms were significantly associated with continuous blood pressure levels across studies in trans-ethnic meta-analyses, the results were heterogeneous across studies and for SBP versus DBP. Indeed, directions of the PTSD main effect varied across cohorts, although significant, yet small, main effects were observed in the overall trans-ethnic meta-analyses and were driven by the largest cohort included: UKBB. Interestingly, greater PTSD symptoms were associated with lower SBP and higher DBP levels in trans-ethnic meta-analyses of all cohorts. In particular, the SBP finding was contrary to our hypotheses given the literature documenting associations between PTSD and elevated blood pressure levels and hypertension (Schnurr et al., 2000; Kibler et al., 2009; Sumner et al., 2016; Burg et al., 2017; Edmondson et al., 2018; Howard et al., 2018). However, when excluding the results of UKBB in trans-ethnic and European ancestry meta-analyses, there was a nominally significant association of greater PTSD symptoms with higher SBP levels, and no significant association was observed for DBP. SBP and DBP exhibit different age-related changes over the lifespan; whereas SBP increases continuously with age, DBP has been found to decrease after 50–60 years of age (Franklin et al., 1997). A number of studies suggest that SBP may be a superior predictor of cardiovascular morbidity and mortality compared to DBP (Kannel et al., 1971; Rutan et al., 1989; Borghi et al., 2003). Furthermore, SBP – and not DBP – is incorporated into various global CVD risk algorithms, such as the Framingham Risk Score and Atherosclerotic CVD Risk Calculator (D’Agostino et al., 2008; Muntner et al., 2014).

The disparate results for associations of PTSD symptoms with blood pressure that were obtained when including versus excluding UKBB may be due, in part, to particular aspects of that cohort’s study design. In UKBB, only six symptoms of PTSD were assessed using self-report, which would be expected to limit the variance in PTSD symptoms in this cohort. In addition, no information on antihypertensive medication was available, which would be expected to result in artificially lowered blood pressure readings for those taking such medications. These study characteristics may explain some differences in findings compared to other studies that comprehensively measured symptoms of PTSD and included information on antihypertensive medication use, allowing for adjustment of estimated blood pressure (Tobin et al., 2005). Nevertheless, together these findings suggest that the link between PTSD and high blood pressure may not be robust or may be subject to substantial individual difference factors that we were not able to study as moderators of the PTSD and blood pressure associations. Similarly, in a meta-analysis of associations between PTSD, heart rate, and blood pressure, the effects for blood pressure – although statistically significant – were smaller than those observed for heart rate (Buckley and Kaloupek, 2001). Findings from the epidemiological literature also suggest that the link between PTSD and high blood pressure may be weaker than those with CVD events and diagnoses, such as myocardial infarction, stroke, and heart disease (Sumner et al., 2015, 2016; El-Gabalawy et al., 2018). Some research also suggests that certain PTSD symptom dimensions (e.g., fear-related symptoms) may be more strongly related to high blood pressure than others (Sumner et al., 2020). In future research, a more nuanced approach that includes deep phenotyping of both PTSD and cardiovascular metrics and that examines potential moderators may be needed to better understand how PTSD relates to blood pressure.

Unlike prior research suggesting that PTSD symptom severity may be most strongly related to cardiometabolic risk among those individuals with greater genetic risk for obesity (Wolf et al., 2017), we did not find significant interactions of PTSD and PGS with blood pressure levels in meta-analyses. Thus, the genetic contributions to blood pressure and any PTSD-related contributions to blood pressure were independent of each other, rather than synergistic. Notably, in these models, we accounted for relevant covariates (i.e., age and sex) and interactions of these covariates with our measures of PTSD symptoms and blood pressure PGS. Therefore, it is unlikely that characteristics such as age were obscuring a potential interaction of PTSD symptoms with PGS. However, due to variability in the measures collected across studies, we were unable to examine the influence of other factors such as cumulative trauma burden, time since trauma, or chronicity of PTSD. In the current investigation, we focused on SBP and DBP rather than other metrics like heart rate because these were the most common cardiovascular measures across studies of the PGC-PTSD Physical Health Working Group. However, it is of interest to leverage this model in future work in an effort to better understand risk for adverse physical health consequences among individuals who experience negative psychiatric sequelae of trauma. In particular, this approach may hold promise for informing screening efforts for chronic disease prevention. Given that associations of PTSD with chronic disease outcomes, including CVD, are complex and multifactorial, comprehensive consideration of multiple disease-related factors may be needed to better elucidate risk.

Our findings need to be considered in light of several limitations. First, although we conducted trans-ethnic meta-analyses across 11 different studies, there was variability in the nature of samples (e.g., military vs. community-based samples) and in the measures of PTSD (e.g., interview- vs. questionnaire-based assessments) and blood pressure (e.g., in protocols and monitors). Including a variety of studies increases the external validity but also contributed to the heterogeneity that we observed. Furthermore, this heterogeneity precluded our ability to engage in deep phenotyping and made it difficult to determine if results were due to differences in sample characteristics or methodological factors. Although we considered secondary stratified analyses in military and community-based cohorts to examine whether this study characteristic might explain some of the heterogeneity we observed in overall analyses, it is worth noting that cohort type overlapped with other study factors (e.g., military cohorts were all- or predominantly male and several community-based cohorts were predominantly female). Additionally, it is possible that community-based cohorts included participants who served in the military but did not collect this information and/or that military cohorts included participants who experienced non-military trauma. Second, despite the large sample size, it is possible that we were statistically underpowered to detect PTSD × Blood Pressure PGS interactions. Future research with larger sample sizes is needed. Third, although we focused in particular on individuals of European and African ancestry in the current investigation given the composition of our cohorts, additional work that incorporates individuals from a variety of additional ancestry groups is needed. Fourth, even though we accounted for antihypertensive medication as recommended by Tobin et al. (2005) for most studies in the current analyses, some studies did not assess medication use, and we were unable to determine adherence to these medications even when it was assessed. However, in meta-analyses restricted to those studies that contributed medication-adjusted blood pressure values, we found similar results. Furthermore, previous studies have shown that certain classes of antihypertensive medications (e.g., angiotensin-converting enzyme inhibitors and angiotensin II receptor blockers) are associated with lower PTSD symptoms in trauma-exposed individuals (Khoury et al., 2012; Seligowski et al., 2021), and that the effects of these antihypertensive medications may be dependent on genetic liability (Nylocks et al., 2015). Despite these limitations, our study has several strengths, including generating PGS from a large trans-ethnic GWAS of blood pressure, utilizing continuous measures of PTSD symptom severity, and conducting team science trans-ethnic meta-analyses. Given that much of genetics research has traditionally focused on individuals of European ancestry, which contributes to disparities in understanding of health-relevant factors for individuals from different backgrounds, our study provides an approach for reducing this problem and increasing representation of diverse ancestries.

## Conclusion

Posttraumatic stress disorder has been linked to adverse cardiovascular health, and research suggests that shared genetic factors may explain, in part, this association (Sumner et al., 2017; Nievergelt et al., 2019). In the current trans-ethnic meta-analyses of over 72,000 European and African ancestry individuals, PTSD symptoms were associated with SBP and DBP levels but in heterogeneous ways and with small effect sizes. Moreover, although underlying PGS significantly predicted blood pressure levels, this genetic metric did not modify associations of PTSD symptoms with SBP or DBP. Further research is needed to better understand the extent to which PTSD is associated with high blood pressure and how genetic factors might play a role in influencing cardiovascular risk. In addition, future studies merit further investigation of the role of environment and context, along with genetics and PTSD symptoms. In examining determinants of health, social determinants, health behaviors, and access to quality medical care are thought to have a larger impact than heredity, with the largest influence being social factors (Tarlov, 1999; Galea et al., 2011). Thus, it is of interest for future studies to adopt a biopsychosocial approach when considering risk for high blood pressure in individuals who have experienced trauma.

## Data Availability Statement

Access to individual-level data for available datasets may be requested through the PGC Data Access Portal at https://www.med.unc.edu/pgc/shared-methods/data-access-portal/. The UKBB data can be obtained by applying here: https://www.ukbiobank.ac.uk/enable-your-research. Other data that contributed to the findings of this study may be available from study Principal Investigators upon request.

## Ethics Statement

The studies involving human participants were reviewed and approved by the Institutional Review Boards of Boston University, Duke University Medical Center, Durham, NC VA, Emory University, Grady Memorial Hospital, Greenslopes Private Hospital, Puget Sound VA Healthcare System, Queensland University of Technology, Stellenbosch University, the Department of Veterans Affairs, University Hospitals, University of California, San Diego, VA Boston Healthcare System, and VA San Diego. The patients/participants provided their written informed consent to participate in this study.

## Author Contributions

JAS, EW, CN, AM, and MWL conceptualized the study and design. LA, OA, AA-K, DB, JB, BB, GB, JC, AD, MD, NF, CF, MG, CG, GG, MH, SH, TJ, NK, WK, BL, AL, MWL, MJL, JM-K, AM, MRM, ReM, DM, RM, VM, WM, MWM, CM, CN, MP, KR, VR, AR, BR, PR-B, SS, AS, JSS, LV, JV, RY, and LZ assisted with obtaining funding for the included studies, and data collection and preparation. AM conducted the analyses, with assistance from MWL and MG. JAS prepared the first draft of the manuscript. AM, VM, AR, and EW provided edits as members of a writing group. All authors reviewed the manuscript, provided feedback, and approved the manuscript for submission.

## Conflict of Interest

The authors declare that the research was conducted in the absence of any commercial or financial relationships that could be construed as a potential conflict of interest.

## Footnotes

1 We examined the genetic correlations of PTSD with SBP and DBP by uploading PTSD GWAS summary statistics from Nievergelt et al. (2019) to LD Hub; GWAS summary statistics for SBP and DBP from UKBB are available on this site. Although statistically significant, the genetic correlation of PTSD with SBP was small (*r*_*g*_ = 0.10, *p* = 4.0E-4); the genetic correlation of PTSD with DBP was not statistically significant (*r*_*g*_ = 0.02, *p* = 0.56). These findings suggest that, by testing PTSD Symptom × Blood Pressure PGS interactions, we were examining Gene × Environment interactions and not environmental factors that were confounded by shared genetics.

2 Results of interaction models were similar when including vs. excluding interactions of all covariates with PTSD symptoms and blood pressure PGS. Thus, we present the results of interaction models with these covariate interactions included per the recommendation of Keller (2014).
