# Supplementary material for: Examining Individual and Synergistic Contributions of PTSD and Genetics to Blood Pressure: A Trans-Ethnic Meta-Analysis
Source: Front Neurosci. 2021 Jun 23;15:678503. doi: 10.3389/fnins.2021.678503 (PMC8262489; doi:10.3389/fnins.2021.678503)
Supplement: Supplementary file 1 [file Data_Sheet_1.docx]

Supplementary Material

# Supplementary Data

**Participating Studies and Methods**

The following studies were included in the current investigation, listed with the official name of each study followed by an abbreviation. As all studies were included in a previously published genome-wide association study of posttraumatic stress disorder (PTSD) from the PGC-PTSD consortium (Nievergelt et al., 2019), we also include the study number that was used to identify each cohort in the Supplementary Data for those analyses to facilitate cross-referencing.

**Study: CHOICE (FEEN; #37)**

See references for details (Jerud et al., 2014;Keller et al., 2014;Bedard-Gilligan et al., 2015;Echiverri-Cohen et al., 2016;Jerud et al., 2016;Post et al., 2016;Bedard-Gilligan et al., 2017;Clifton et al., 2017;Cooper et al., 2017;Jerud et al., 2017). Potentially traumatic events were identified using the standard trauma interview (Resnick et al., 1993). The PTSD Symptom Scale–Interview (PSS-I) was used to assess PTSD over the prior two weeks for the trauma of interest by postdoctoral or graduate level assessors trained to reliability (First and Gibbon, 2004). The Structured Clinical Interview for the *Diagnostic and Statistical Manual for Mental Disorders*, 4^th^ edition (*DSM-IV*) (SCID-IV) was used to assess lifetime PTSD (not current) for a trauma not the focus of treatment by postdoctoral or graduate level assessors trained to reliability (Foa et al., 1993). Respondents were considered to have a current diagnosis if on the PSS-I they met symptom-level *DSM-IV* diagnostic criteria. The PSS-I also provides PTSD symptom severity. Blood pressure was assessed using automatic electronic blood pressure cuffs supervised by a graduate or postdoctoral level clinician. Blood pressure was collected at the first therapy session before therapy commenced. DNA for GWAS analysis was isolated from blood. The Institutional Review Board of University Hospitals approved this study.

**Study: D-cycloserine Study (DCS; #38)**

See references for details (Rothbaum et al., 2014;Norrholm et al., 2016). This study examined the effectiveness of virtual reality exposure augmented with D-cycloserine or alprazolam, compared with placebo, in reducing PTSD due to military trauma. After an introductory session, five sessions of virtual reality exposure were augmented with D-cycloserine (50 mg) or alprazolam (0.25 mg) in a double-blind, placebo-controlled randomized clinical trial for 156 Iraq and Afghanistan war veterans with PTSD. PTSD symptoms significantly improved from pre- to posttreatment across all conditions and were maintained at 3, 6, and 12 months, such that a six-session virtual reality treatment was associated with reduction in PTSD diagnoses and symptoms. There were no overall differences in symptoms between D-cycloserine and placebo at any time. Alprazolam and placebo differed significantly on the Clinician-Administered PTSD Scale (CAPS) for *DSM-IV* (Blake et al., 1995) score at posttreatment and PTSD diagnosis at 3 months posttreatment; the alprazolam group showed a higher rate of PTSD (82.8%) than the placebo group (47.8%). Between-session extinction learning was a treatment-specific enhancer of outcome for the D-cycloserine group only. At posttreatment, the D-cycloserine group had the lowest cortisol reactivity and smallest startle response during virtual reality scenes.

For the current study, PTSD symptoms were measured with the PTSD Symptom Scale, Self-report version (PSS-SR; Foa et al., 1997). Blood pressure was collected using a standard manual pressure cuff by a trained research coordinator or postdoctoral fellow at the pre-treatment assessment. DNA for GWAS analysis was isolated from saliva. The Institutional Review Board of Emory University approved this study.

**Study: Gallipoli Medical Research Foundation-Queensland University of Technology (GMRF-QUT; #55)**

See reference for details (Mehta et al., 2017). Potentially traumatic events were identified using Criterion A events. The CAPS for *DSM-5* (CAPS-5) was used to assess PTSD over the prior two weeks and lifetime by clinical psychologists (Weathers et al., 2013a). Respondents were considered to have a current diagnosis if CAPS-5 criteria were met. The CAPS-5 also calculates PTSD symptom severity. Blood pressure was measured via standard practice using a WelchAllyn machine (NIBP/SPO2-MONITOR, 6000 XXXX Series, BME6464) by trained hospital staff with participants seated or lying supine. The equipment for measuring vital signs was calibrated annually or as required. Use of antihypertensive medication was assessed during a medical history interview. DNA for GWAS analysis was isolated from peripheral blood. Ethics approval for the project was obtained from the Department of Veterans’ Affairs, Greenslopes Private Hospital, and Queensland University of Technology Human Research Ethics Committees. This study was carried out in accordance with the Code of Ethics of the World Medical Association (Declaration of Helsinki).

**Study: Grady Trauma Project (GTP; #47)**

See reference for details (Binder et al., 2008). The modified PTSD Symptom Scale (mPSS), a psychometrically valid 17-item self-report scale assessing PTSD symptomatology over the prior 2 weeks, was used to assess PTSD (Falsetti et al., 1993). Consistent with prior literature, the PSS frequency items (0 indicates not at all to 3 indicates ≥5 times a week) were combined to obtain a continuous measure of PTSD symptom severity. Participants also underwent a physician-administered examination where blood pressure was collected once by trained nursing staff at the Grady Hospital Clinical Research Center (Khoury et al., 2012;Nylocks et al., 2015). Prescription antihypertensive medications were also recorded at this examination by study physicians based on self-report (Khoury et al., 2012;Nylocks et al., 2015), including diuretics, calcium channel blockers, angiotensin converting enzyme inhibitors, and adrenergic blocking agents. The Institutional Review Boards of Emory University School of Medicine and Grady Memorial Hospital approved this study.

**Study: Hostility, PTSD, and Physical Health Risk Factors (HOST; #26)**

See reference for details (Oddone et al., 2015). Participants included U.S. civilians and veterans between 18-39 years of age. PTSD diagnosis and symptom severity were assessed with the CAPS for *DSM-IV* (Blake et al., 1995) by trained interviewers. The CAPS is a 17-item semi-structured interview used to assess *DSM-IV* criteria for PTSD that was administered to both cases and controls. In addition to diagnosis, the CAPS yields a total PTSD symptom severity score, which was used to assess current PTSD symptom severity in the present analyses.

Direct measurements were obtained for systolic and diastolic blood pressure (SBP and DBP). Blood pressure data were measured during the third study visit, approximately one week after the CAPS had been completed. SBP and DBP measurements were obtained in triplicate after participants had been sitting for 5 minutes according to American Heart Association guidelines, using a Datascope Accutorr Plus blood pressure monitor. The first two blood pressure measurements were taken in the right arm, whereas the last was taken in the left arm. The means of the three readings for SBP and DBP were used in the present analyses. Medications, including antihypertensive medications, were recorded and classified based on our previous work involving the input and consensus of our team psychiatrists.

The research was reviewed and approved by the Institutional Review Boards at the Durham, NC VA and Duke University Medical Centers.

**Study: Marine Resiliency Study (MRS: #1)**

See references for details (Baker et al., 2012;Nievergelt et al., 2015). Participants were recruited for a prospective PTSD study with longitudinal follow-up (pre- and post-exposure to combat stress) of U.S. Marines bound for deployment to Iraq or Afghanistan. PTSD was diagnosed up to 3 times, once before deployment and 3 and/or 6 months post-deployment. PTSD symptoms were assessed using a structured diagnostic interview, the CAPS, and PTSD diagnosis followed the *DSM-IV* criteria (Blake et al., 1995). All participants included in this study met the *DSM-IV* Criterion A1 event. For participants assessed at multiple timepoints, the post-deployment timepoint with the highest CAPS score was used.

SBP and DBP were collected using the DynaPulse oscillometric brachial cuff (PulseMetric, Vista, California), matching the timepoint with the highest CAPS score (Brinton et al., 1997). We corrected output SBP values using previously validated adjustments to data (Chio et al., 2011). Trained lab personnel affixed seated MRS participants with the brachial DynaPulse cuff, which then acquired the data on the outstretched dominant arm. Traits were measured 3 times, at 3-minute intervals, and then measurements were averaged. Our analysis is based upon these averaged estimates. Use of antihypertensive medication was assessed at the time of hemodynamic assessment.

Genomic DNA was prepared from blood leukocytes and genotyping was carried out by Illumina (http://www.illumina.com/) using the HumanOmniExpressExome (HOEE) array with 951,117 loci and by RUCDR (http://www.rucdr.org) using the HOEE array with 967,537 loci. The study was approved by the University of California San Diego Institutional Review Board and the VA San Diego Institutional Review Board, and all participants provided written informed consent to participate.

**Study: Optimizing Treatment for PTSD (OPT; #37)**

Potentially traumatic events were identified using the standard trauma interview (Resnick et al., 1996). The PSS-I was used to assess PTSD over the prior two weeks for the trauma of interest by postdoctoral and graduate level assessors trained to reliability (Foa et al., 1993). The SCID-IV was used to assess lifetime PTSD (not current) for a single trauma not the focus of treatment (First and Gibbon, 2004). The PSS-I also provides PTSD symptom severity. Blood pressure was assessed using automatic electronic blood pressure cuffs supervised by a graduate or postdoctoral level clinician. Blood pressure was collected at the first therapy session before therapy commenced. For the subset of participants receiving sertraline, active medications were assessed at the randomization by a board-certified psychiatrist. DNA for GWAS analysis was isolated from blood. The Institutional Review Board of University Hospitals approved this study.

**Study: Shared Roots Study (SHRS; #46)**

SHRS or “Understanding the SHARED ROOTS of Neuropsychiatric Disorders and Modifiable Risk Factors for Cardiovascular Disease” was a matched case-control study examining the factors that contribute to the comorbidity of metabolic syndrome and neuropsychiatric disorders (van den Heuvel et al., 2020). Potentially traumatic events were identified using the Life Events Checklist for *DSM-5* (LEC-5; Weathers et al., 2013b). The CAPS-5 (Weathers et al., 2013a) was administered by clinicians to assess PTSD over the prior month. The CAPS-5 and the PTSD Checklist for *DSM-5* (PCL-5; Weathers et al., 2013c) were both administered to assess PTSD symptom severity. Lifetime diagnosis of PTSD was not assessed for in this study. Physical measurements were performed according to the World Health Organization STEPS guideline (World Health Organization, 2005). Blood pressure was measured after the person had been seated for at least 15 minutes using a digital automated blood pressure monitor. Three blood pressure readings were taken three minutes apart. The mean of the second and third readings was used to calculate the mean SBP and DBP. Blood pressure was measured within 24-96 hours of assessments for PTSD. Use of antihypertensive medication was also assessed. The Institutional Review Board of Stellenbosch University approved this study.

**Study: Translational Research Center for TBI and Stress Disorders at VA Boston-National Center for PTSD (TRACTS; #32)**

This cohort was comprised of veterans from the Translational Research Center for TBI and Stress Disorders, a VA RR&D Traumatic Brain Injury Center of Excellence at VA Boston Healthcare System (TRACTS). From TRACTS, 200 white non-Hispanic cases and 112 controls passed ancestry filters based on SNPweights and were included in the analysis. Participants were administered the CAPS for *DSM-IV* (Blake et al., 1995), a 30-item structured diagnostic interview that assesses the frequency and severity of the 17 *DSM-IV* PTSD symptoms, 5 associated features and functional impairment, and both current and lifetime PTSD symptoms. Two seated blood pressure readings were obtained (about two minutes apart) using an automatic sphygmometer and averaged. Antihypertensive medication use was based on veteran self-report (and confirmed in the medical record when need be). The genotyping, quality control, filtering and imputation for these cohorts has been described in detail elsewhere (Logue et al., 2013;Sadeh et al., 2016). Briefly, genotyping was performed using the Illumina HumanOmni2.5-8 microarrays (Illumina, San Diego, CA). Imputation of non-genotyped SNPs was performed using IMPUTE2 (Howie et al., 2009;Marchini and Howie, 2010;Howie et al., 2011;Howie et al., 2012) and 1000 Genomes phase 1 reference data (Genomes Project et al., 2012). Principal components were generated by the program EIGENSTRAT (Price et al., 2006) based on 100,000 SNPs. These studies were performed under the oversight of the appropriate VA health care facilities Institutional Review Boards.

**Study: UK Biobank (UKBB; #60)**

The UK Biobank is an epidemiological resource assessing a range of health-related phenotypes in approximately 500,000 British individuals who were recruited between the ages of 40 and 70 (Allen et al., 2014). Genome-wide genotype data are available on all participants, as well as a broad range of health phenotypes. Data from an online follow-up questionnaire assessing common mental health traits, including questions designed to screen for PTSD, were available on 157,366 individuals (Davis et al., 2020).

PTSD phenotypes were derived from the mental health online follow-up of the UK Biobank (Resource 22 on http://biobank.ctsu.ox.ac.uk). Participants were asked six questions derived from the brief civilian version of the PTSD Checklist Screener (Wilkins et al., 2011) assessing PTSD symptoms in the prior month. Questions comprised three initial questions related to avoidance of activities, disturbing thoughts, and feeling upset, and three further questions related to feeling distant, feeling irritable and having trouble concentrating (UK Biobank fields 20494-20498, 20508). Each item was scored on a five-point scale according to the amount of concern caused by that item in the past month (1="Not at all" to 5="Extremely"). The final item—trouble concentrating—was drawn from an equivalent item from the Patient Health Questionnaire-9 (PHQ-9) depression questionnaire and was scored on a four-point scale according to frequency of difficulties (1="Not at all" and 4="Nearly every day"). All items were summed to yield a total symptom score. A lifetime trauma exposure measure was derived from questions in the mental health online follow-up that related to common triggers of PTSD (Davis et al., 2018). These questions asked if participants had ever: experienced combat; had a life-threatening accident; been diagnosed with a life-threatening illness; been a victim of a physically violent crime; been a victim of sexual assault; or witnessed a sudden violent death. Responses were combined to a single variable capturing any report of trauma exposure versus no report, and analyses were restricted to participants who had experienced trauma during their lifetime.

Resting blood pressure was measured at Assessment Centers of the UK Biobank. Participants were seated with their feet parallel and flat on the floor, toes pointing forward. The left arm was used for blood pressure measurement unless this was impractical, in which case the right arm was used. An appropriately sized cuff was placed on the upper arm, and participants were then instructed to place their arm on a desk top, at level with their heart, and to breathe in and out slowly five times in a relaxed manner. SBP and DBP were then measured with an Omron 705 IT electronic blood pressure monitor. A second blood pressure measurement was collected after at least one minute had elapsed since the last reading. The two SBP and DBP readings were each averaged.

Genetic data for analyses were obtained from the full release of the UK Biobank data (*N*=487,410; Bycroft et al., 2018). Individuals were removed if this was recommended by the UK Biobank for unusual levels of missingness or heterozygosity; if call rate was < 98% on genotyped SNPs; if they were related to another individual in the dataset (KING *r* < .044, equivalent to removing up to third-degree relatives inclusive); and if the phenotypic and genotypic gender information was discordant (X-chromosome homozygosity (FX) < 0.9 for phenotypic males, FX > 0.5 for phenotypic females). Removal of relatives was performed using a greedy algorithm, which minimizes exclusions (for example, by excluding the child in a mother-father-child trio). All analyses were limited to individuals of White Western European ancestry, as defined by 4-means clustering on the first two genetic principal components provided by the UK Biobank (Warren et al., 2017). Principal components analysis was also performed on the European-only subset of the data using the software package flashpca2 (Abraham et al., 2017). After quality control, individuals were excluded from analysis if they did not complete the mental health online questionnaire (*N*=126,522).

Genetic analyses used imputed variants provided by the UK Biobank (Bycroft et al., 2018). Autosomal genotype data from two highly-overlapping custom genotyping arrays (covering ~800,000 markers) underwent centralised quality control to remove genotyping errors before being imputed in a two-stage imputation to the Haplotype Reference Consortium (HRC) and UK10K (for rarer variants not present in the HRC) reference panels (UK10K Consortium, 2015;McCarthy et al., 2016;Bycroft et al., 2018). In addition, variants for analysis were limited to common variants (minor allele frequency > 0.01) imputed with higher confidence (IMPUTE INFO metric > 0.4). In addition, only variants that were directly genotyped or that were imputed from the HRC were included (McCarthy et al., 2016).

**Study: Vietnam Era Twin Study of Aging (VETSA; #24)**

See references for details (Kremen et al., 2006;Kremen et al., 2013). Potentially traumatic events were identified using the Combat Exposure Index (Janes et al., 1991) and the Diagnostic Interview Schedule Version III-Revised (DIS-III-R; Robins et al., 1981) The Vietnam Era Twin Registry PTSD scale (Goldberg et al., 1990; administered at average age 38) was used to assess PTSD symptoms over the past 6 months and the PCL-Civilian version for *DSM-IV* (Weathers et al., 1994; administered at average age 62) was used to assess PTSD over the prior month. These two instruments correlate 0.90 when administered at the same time (Magruder et al., 2015). The 17-item PCL calculated PTSD symptom severity. Each response was rated on a 1-5 scale (from “Not at all” to “Extremely”).

Blood pressure was measured both in the morning and afternoon at an exact point in the order of testing in person, the day of testing, using a LifeSource Automatic Blood Pressure Monitor Model UA-789. The same procedure was followed for the two morning assessments and two afternoon assessments. Blood pressure was measured with the participant seated. The tester recorded the time of measurement, arm used for measurement, cuff size, and cuff snugness. After the cuff was put on the upper arm, testers left the room for 5 minutes after instructing the participant to sit quietly for 5 minutes and not to cross their arms or legs. The tester remained outside the room to ensure quiet. When the tester returned to the room, the first measurement was taken, and SBP and DBP were recorded. SBP and DBP were then measured again one minute after the first reading. Outliers (SBP > 220; DBP < 50 or > 120) were examined as part of data cleaning. Participants with fewer than three SBP or DBP measures were excluded. Two scores were provided for this study: the average of the four SBP measures and average of the four DBP measures. As part of the Medical History Interview on the day of testing, participants listed prescription and over-the-counter medications that they were currently taking (medications were brought in a bag to the testing site). These were later assigned 3-letter medication codes by the tester. For some medications relevant to VETSA papers, project physicians identified clusters of medications for particular purposes (e.g., depression, hypertension, diabetes) and the prescription drug data processed to identify these. Participants are coded as 1=any (vs. 0=none) if they reported currently taking any antihypertensive medication.

DNA for GWAS analysis was isolated from blood. Genotyping was performed by deCODE Genetics, Reykjavik, Iceland. The Institutional Review Boards of the University of California, San Diego, Boston University, and the Puget Sound VA Healthcare System approved this study.

**References:**

Abraham, G., Qiu, Y., and Inouye, M. (2017). FlashPCA2: principal component analysis of Biobank-scale genotype datasets. Bioinformatics 33, 2776-2778.

Allen, N.E., Sudlow, C., Peakman, T., and Collins, R. (2014). UK biobank data: come and get it. Sci. Transl. Med. 6, 224ed4.

Baker, D.G., Nash, W.P., Litz, B.T., Geyer, M.A., Risbrough, V.B., Nievergelt, C.M., O'Connor, D.T., Larson, G.E., Schork, N.J., Vasterling, J.J., Hammer, P.S., Webb-Murphy, J.A., and Team, M.R.S. (2012). Predictors of risk and resilience for posttraumatic stress disorder among ground combat Marines: methods of the Marine Resiliency Study. Prev. Chronic Dis. 9, E97.

Bedard-Gilligan, M., Duax Jakob, J.M., Doane, L.S., Jaeger, J., Eftekhari, A., Feeny, N., and Zoellner, L.A. (2015). An investigation of depression, trauma history, and symptom severity in individuals enrolled in a treatment trial for chronic PTSD. J. Clin. Psychol. 71, 725-740.

Bedard-Gilligan, M., Zoellner, L.A., and Feeny, N.C. (2017). Is trauma memory special? Trauma narrative fragmentation in PTSD: effects of treatment and response. Clin. Psychol. Sci. 5, 212-225.

Binder, E.B., Bradley, R.G., Liu, W., Epstein, M.P., Deveau, T.C., Mercer, K.B., Tang, Y., Gillespie, C.F., Heim, C.M., Nemeroff, C.B., Schwartz, A.C., Cubells, J.F., and Ressler, K.J. (2008). Association of FKBP5 polymorphisms and childhood abuse with risk of posttraumatic stress disorder symptoms in adults. JAMA. 299, 1291-1305.

Blake, D.D., Weathers, F.W., Nagy, L.M., Kaloupek, D.G., Gusman, F.D., Charney, D.S., and Keane, T.M. (1995). The development of a clinician-administered PTSD scale. J. Trauma. Stress 8, 75-90.

Brinton, T.J., Cotter, B., Kailasam, M.T., Brown, D.L., Chio, S.S., O'connor, D.T., and Demaria, A.N. (1997). Development and validation of a noninvasive method to determine arterial pressure and vascular compliance. Am. J. Cardiol. 80, 323-330.

Bycroft, C., Freeman, C., Petkova, D., Band, G., Elliott, L.T., Sharp, K., Motyer, A., Vukcevic, D., Delaneau, O., O'Connell, J, Cortes, A., Welsh, S., Young, A., Effingham, M., McVean, G., Leslie, S., Allen, N., Donnelly, P., and Marchini, J. (2018). The UK Biobank resource with deep phenotyping and genomic data. Nature 562, 203-209.

Chio, S.S., Urbina, E.M., Lapointe, J., Tsai, J., and Berenson, G.S. (2011). Korotkoff sound versus oscillometric cuff sphygmomanometers: comparison between auscultatory and DynaPulse blood pressure measurements. J. Am. Soc. Hypertens. 5, 12-20.

Clifton, E.G., Feeny, N.C., and Zoellner, L.A. (2017). Anger and guilt in treatment for chronic posttraumatic stress disorder. J. Behav. Ther. Exp. Psychiatry 54, 9-16.

UK10K Consortium (2015). The UK10K project identifies rare variants in health and disease. Nature 526, 82.

Cooper, A.A., Kline, A.C., Graham, B., Bedard-Gilligan, M., Mello, P.G., Feeny, N.C., and Zoellner, L.A. (2017). Homework “dose,” type, and helpfulness as predictors of clinical outcomes in prolonged exposure for PTSD. Behav. Ther. 48, 182-194.

Davis, K.A.S., Coleman, J.R.I., Adams, M., Allen, N., Breen, G., Cullen, B., Dickens, C., Fox, E., Graham, N., Holliday, J., Howard, L.M., John, A., Lee, W., McCabe, R., McIntosh, A., Pearsall, R., Smith, D.J., Sudlow, C., Ward, J., Zammit, S., and Hotopf, M. (2020). Mental Health in UK Biobank–development, implementation and results from an online questionnaire completed by 157 366 participants: a reanalysis. BJPsych. Open 6, e18.

Echiverri-Cohen, A., Zoellner, L.A., Gallop, R., Feeny, N., Jaeger, J., and Bedard-Gilligan, M. (2016). Changes in temporal attention inhibition following prolonged exposure and sertraline in the treatment of PTSD. J. Consult. Clin. Psychol. 84, 415-426.

Falsetti, S.A., Resnick, H.S., Resick, P.A., and Kilpatrick, D.G. (1993). The modified PTSD symptom scale: a brief self-report measure of posttraumatic stress disorder. Behav. Ther. 16, 161-162.

First, M.B., and Gibbon, M. (2004). “The Structured Clinical Interview for DSM-IV Axis I Disorders (SCID-I) and the Structured Clinical Interview for DSM-IV Axis II Disorders (SCID-II)” in Comprehensive Handbook of Psychological Assessment, Vol. 2, eds. M.J. Hilsenroth and D.L. Segal (Hoboken, NJ; John Wiley & Sons Inc), 134-143.

Foa, E., Cashman, L., Jaycox, L., and Perry, K. (1997). The validation of a self-report measure of PTSD: the Posttraumatic Diagnostic Scale. Psychol. Assess. 9, 445-451.

Foa, E.B., Riggs, D.S., Dancu, C.V., and Rothbaum, B.O. (1993). Reliability and validity of a brief instrument for assessing post‐traumatic stress disorder. J. Trauma. Stress 6, 459-473.

Genomes Project, C., Abecasis, G.R., Auton, A., Brooks, L.D., Depristo, M.A., Durbin, R.M., Handsaker, R.E., Kang, H.M., Marth, G.T., and Mcvean, G.A. (2012). An integrated map of genetic variation from 1,092 human genomes. Nature 491, 56-65.

Goldberg, J., Eisen, S.A., Trub, W.R., and Henderson, W.G. (1990). A twin study of the effects of the Vietnam conflict on alcohol drinking patterns. Am. J. Public Health 80, 570-574.

Howie, B., Fuchsberger, C., Stephens, M., Marchini, J., and Abecasis, G.R. (2012). Fast and accurate genotype imputation in genome-wide association studies through pre-phasing. Nat. Genet. 44, 955-959.

Howie, B., Marchini, J., and Stephens, M. (2011). Genotype imputation with thousands of genomes. G3 1, 457-470.

Howie, B.N., Donnelly, P., and Marchini, J. (2009). A flexible and accurate genotype imputation method for the next generation of genome-wide association studies. PLoS Genet. 5, e1000529.

Janes, G.R., Goldberg, J., Eisen, S.A., and True, W.R. (1991). Reliability and validity of a combat exposure index for Vietnam era veterans. J. Clin. Psychol. 47, 80-86.

Jerud, A.B., Farach, F.J., Bedard‐Gilligan, M., Smith, H., Zoellner, L.A., and Feeny, N.C. (2017). Repeated trauma exposure does not impair distress reduction during imaginal exposure for posttraumatic stress disorder. Depress. Anxiety 34, 671-678.

Jerud, A.B., Pruitt, L.D., Zoellner, L.A., and Feeny, N.C. (2016). The effects of prolonged exposure and sertraline on emotion regulation in individuals with posttraumatic stress disorder. Behav. Res. Ther. 77, 62-67.

Jerud, A.B., Zoellner, L.A., Pruitt, L.D., and Feeny, N.C. (2014). Changes in emotion regulation in adults with and without a history of childhood abuse following posttraumatic stress disorder treatment. J. Consult. Clin. Psychol. 82, 827-827.

Keller, S.M., Feeny, N.C., and Zoellner, L.A. (2014). Depression sudden gains and transient depression spikes during treatment for PTSD. J. Consult. Clin. Psychol. 82, 102-111.

Khoury, N.M., Marvar, P.J., Gillespie, C.F., Wingo, A., Schwartz, A., Bradley, B., Kramer, M., and Ressler, K.J. (2012). The renin-angiotensin pathway in posttraumatic stress disorder: angiotensin-converting enzyme inhibitors and angiotensin receptor blockers are associated with fewer traumatic stress symptoms. J. Clin. Psychiatry 73, 849-855.

Kremen, W.S., Franz, C.E., and Lyons, M.J. (2013). VETSA: the Vietnam Era Twin Study of Aging. Twin Res. Hum. Genet. 16, 399-402.

Kremen, W.S., Thompson-Brenner, H., Leung, Y.M., Grant, M.D., Franz, C.E., Eisen, S.A., Jacobson, K.C., Boake, C., and Lyons, M.J. (2006). Genes, environment, and time: the Vietnam Era Twin Study of Aging (VETSA). Twin Res. Hum. Genet. 9, 1009-1022.

Logue, M.W., Baldwin, C., Guffanti, G., Melista, E., Wolf, E.J., Reardon, A.F., Uddin, M., Wildman, D., Galea, S., Koenen, K.C., and Miller, M.W. (2013). A genome-wide association study of post-traumatic stress disorder identifies the retinoid-related orphan receptor alpha (RORA) gene as a significant risk locus. Mol. Psychiatry 18, 937-942.

Magruder, K., Yeager, D., Goldberg, J., Forsberg, C., Litz, B., Vaccarino, V., Friedman, M., Gleason, T., Huang, G., and Smith, N. (2015). Diagnostic performance of the PTSD checklist and the Vietnam Era Twin Registry PTSD scale. Epidemiol. Psychiatr. Sci. 24, 415-422.

Marchini, J., and Howie, B. (2010). Genotype imputation for genome-wide association studies. Nat. Rev. Genet. 11, 499-511.

Mccarthy, S., Das, S., Kretzschmar, W., Delaneau, O., Wood, A.R., Teumer, A., Kang, H.M., Fuchsberger, C., Danecek, P., and Sharp, K. (2016). A reference panel of 64,976 haplotypes for genotype imputation. Nat. Genet. 48, 1279.

Mehta, D., Bruenig, D., Carrillo‐Roa, T., Lawford, B., Harvey, W., Morris, C., Smith, A., Binder, E., Young, R.M., and Voisey, J. (2017). Genomewide DNA methylation analysis in combat veterans reveals a novel locus for PTSD. Acta Psychiatr. Scand*.* 136, 493-505.

Nievergelt, C.M., Maihofer, A.X., Klengel, T., Atkinson, E.G., Chen, C.-Y., Choi, K.W., Coleman, J.R., Dalvie, S., Duncan, L.E., Gelernter, J., Levey, D.F., Logue, M.W., Polimanti, R., Provost, A.C., Ratanatharathorn, A., Stein, M.B., Torres, K., Aiello, A.E., Almli, L.M., Amstadter, A.B., Andersen, S.B., Andreassen, O.A., Arbisi, P.A., Ashley-Koch, A.E., Austin, S.B., Avdibegovic, E., Babić, D., Baekvad-Hansen, M., Baker, D.G., Beckham, J.C., Bierut, L.J., Bisson, J.I., Boks, M.P., Bolger, E.A., Børglum, A.D., Bradley, B., Brashear, M., Breen, G., Bryant, R.A., Bustamante, A.C., Byberg-Grauholm, J., Calabrese, J.R., Caldas-De-Almeida, J., Dale, A.M., Daly, M.J., Daskalakis, N.P., Deckert, J., Delahanty, D.L., Dennis, M.F., Disner, S.G., Domschke, K., Dzubur-Kulenovic, A., Erbes, C.R., Evans, A., Farrer, L.A., Feeny, N.C., Flory, J.D., Forbes, D., Franz, C.E., Galea, S., Garrett, M.E., Gelaye, B., Geuze, E., Gillespie, C.F., Goci Uka, A., Gordon, S.D., Guffanti, G., Hammamieh, R., Harnal, S., Hauser, M.A., Heath, A.C., Hemmings, S.M.J., Hougaard, D.M., Jakovljevic, M., Jett, M., Johnson, E.O., Jones, I., Jovanovic, T., Qin, X.-J., Junglen, A.G., Karstoft, K.-I., Kaufman, M.L., Kessler, R.C., Khan, A., Kimbrel, N.A., King, A.P., Koen, N., Kranzler, H.R., Kremen, W.S., Lawford, B.R., Lebois, L.a.M., Lewis, C.E., Linnstaedt, S.D., Lori, A., Lugonja, B., Luykx, J.J., Lyons, M.J., Maples-Keller, J., Marmar, C., Martin, A.R., et al. (2019). International meta-analysis of PTSD genome-wide association studies identifies sex-and ancestry-specific genetic risk loci. Nat. Commun.10, 4558.

Nievergelt, C.M., Maihofer, A.X., Mustapic, M., Yurgil, K.A., Schork, N.J., Miller, M.W., Logue, M.W., Geyer, M.A., Risbrough, V.B., O'connor, D.T., and Baker, D.G. (2015). Genomic predictors of combat stress vulnerability and resilience in U.S. Marines: A genome-wide association study across multiple ancestries implicates PRTFDC1 as a potential PTSD gene. Psychoneuroendocrinology 51, 459-471.

Norrholm, S.D., Jovanovic, T., Gerardi, M., Breazeale, K.G., Price, M., Davis, M., Duncan, E., Ressler, K.J., Bradley, B., Rizzo, A., Tuerk, P.W., and Rothbaum, B.O. (2016). Baseline psychophysiological and cortisol reactivity as a predictor of PTSD treatment outcome in virtual reality exposure therapy. Behav. Res. Ther. 82, 28-37.

Nylocks, K.M., Michopoulos, V., Rothbaum, A.O., Almli, L., Gillespie, C.F., Wingo, A., Schwartz, A.C., Habib, L., Gamwell, K.L., Marvar, P.J., Bradley, B., and Ressler, K.J. (2015). An angiotensin-converting enzyme (ACE) polymorphism may mitigate the effects of angiotensin-pathway medications on posttraumatic stress symptoms. Am. J. Med. Genet. B Neuropsychiatr. Genet. 168B, 307-315.

Oddone, A.E., Dennis, P.A., Calhoun, P.S., Watkins, L.L., Sherwood, A., Dedert, E.A., Green, K.T., Stein, J.N., Dennis, M.F., and Beckham, J.C. (2015). Orthostatic hypotension in young adults with and without posttraumatic stress disorder. Psychol. Trauma 7, 229-233.

Post, L.M., Feeny, N.C., Zoellner, L.A., and Connell, A.M. (2016). Post‐traumatic stress disorder and depression co‐occurrence: Structural relations among disorder constructs and trait and symptom dimensions. Psychol. Psychother. 89, 418-434.

Price, A.L., Patterson, N.J., Plenge, R.M., Weinblatt, M.E., Shadick, N.A., and Reich, D. (2006). Principal components analysis corrects for stratification in genome-wide association studies. Nat. Genet. 38, 904-909.

Resnick, H., Best, C., Kilpatrick, D., Freedy, J., and Falsetti, S. (1996). “Assessment of rape and other civilian trauma-related post-traumatic stress disorder: emphasis on assessment of potentially traumatic events” in Stressful Life Events, ed. T.W. Miller (Madison, WI; International Universities Press), 231-266.

Robins, L.N., Helzer, J.E., Croughan, J., and Ratcliff, K.S. (1981). National Institute of Mental Health Diagnostic Interview Schedule. Its history, characteristics, and validity. Arch. Gen. Psychiatry 38, 381-389.

Rothbaum, B.O., Price, M., Jovanovic, T., Norrholm, S.D., Gerardi, M., Dunlop, B., Davis, M., Bradley, B., Duncan, E.J., Rizzo, A., and Ressler, K.J. (2014). A randomized, double-blind evaluation of D-cycloserine or alprazolam combined with virtual reality exposure therapy for posttraumatic stress disorder in Iraq and Afghanistan War veterans. Am. J. Psychiatry 171, 640-648.

Sadeh, N., Spielberg, J.M., Logue, M.W., Wolf, E.J., Smith, A.K., Lusk, J., Hayes, J.P., Sperbeck, E., Milberg, W.P., Mcglinchey, R.E., Salat, D.H., Carter, W.C., Stone, A., Schichman, S.A., Humphries, D.E., and Miller, M.W. (2016). SKA2 methylation is associated with decreased prefrontal cortical thickness and greater PTSD severity among trauma-exposed veterans. Mol. Psychiatry 21, 357-363.

Van Den Heuvel, L.L., Stalder, T., Du Plessis, S., Suliman, S., Kirschbaum, C., and Seedat, S. (2020). Hair cortisol levels in posttraumatic stress disorder and metabolic syndrome. Stress 23, 577-589.

Warren, H.R., Evangelou, E., Cabrera, C.P., Gao, H., Ren, M., Mifsud, B., Ntalla, I., Surendran, P., Liu, C., and Cook, J.P. (2017). Genome-wide association analysis identifies novel blood pressure loci and offers biological insights into cardiovascular risk. Nat. Genet. 49, 403.

Weathers, F., Blake, D., Schnurr, P., Kaloupek, D., Marx, B., and Keane, T. (2013a). The clinician-administered PTSD scale for DSM-5 (CAPS-5). *Available from www.ptsd.va.gov*.

Weathers, F., Blake, D., Schnurr, P., Kaloupek, D., Marx, B., and Keane, T. (2013b). The life events checklist for DSM-5 (LEC-5). *Available from www.ptsd.va.gov*.

Weathers, F.W., Litz, B., Keane, T., Palmieri, P., Marx, B., and Schnurr, P. (2013c). The PTSD checklist for DSM-5 (PCL-5). *Available from www.ptsd.va.gov*.

Weathers, F.W., Litz, B.T., Huska, J.A., and Keane, T.M. (1994). The PTSD checklist, civilian version. *Available from www.ptsd.va.gov*.

Wilkins, K.C., Lang, A.J., and Norman, S.B. (2011). Synthesis of the psychometric properties of the PTSD checklist (PCL) military, civilian, and specific versions. Depress. Anxiety 28, 596-606.

World Health Organization (2005). “WHO STEPS surveillance manual: the WHO STEPwise approach to chronic disease risk factor surveillance.” World Health Organization. <https://apps.who.int/iris/handle/10665/4337>

**Supplementary Table 1.** Results of sex-specific analyses of main effects of polygenic scores and PTSD symptoms, along with their interaction, on blood pressure in the UK Biobank cohort.

|  | **Systolic Blood Pressure** | | |  | | **Diastolic Blood Pressure** | | |
| --- | --- | --- | --- | --- | --- | --- | --- | --- |
| **Effect** | **Beta (SE)** | ***t*** | ***p*** | |  | **Beta (SE)** | ***t*** | ***p*** |
| PGS main effect |  |  |  | |  |  |  |  |
| Men | 2.81 (0.10) | 29.15 | <1E-20 | |  | 1.26 (0.06) | 21.67 | <1E-20 |
| Women | 3.25 (0.09) | 34.93 | <1E-20 | |  | 1.46 (0.05) | 26.87 | <1E-20 |
| Sex x PGS interaction | -0.42 (0.14) | -3.09 | 2.0E-3 | |  | -0.20 (0.08) | -2.52 | .01 |
|  |  |  |  | |  |  |  |  |
| PTSD symptoms main effect |  |  |  | |  |  |  |  |
| Men | -3.24 (0.74) | -4.39 | 1.1E-5 | |  | 0.10 (0.45) | 0.23 | .82 |
| Women | -1.87 (0.62) | -3.01 | 2.7E-3 | |  | 0.89 (0.36) | 2.48 | .01 |
| Sex x PTSD Symptoms interaction | 1.27 (0.98) | 1.30 | .19 | |  | 0.24 (0.57) | 0.41 | .68 |
|  |  |  |  | |  |  |  |  |
| PTSD Symptoms x PGS interaction |  |  |  | |  |  |  |  |
| Men | -0.65 (0.76) | -0.86 | .39 | |  | -0.17 (0.46) | -0.36 | .72 |
| Women | 0.24 (0.62) | 0.40 | .69 | |  | 0.15 (0.37) | 0.42 | .67 |

*Note.* Models adjusted for the first five ancestry principal components, age, age-squared, sex, and interactions of all covariates with PTSD symptoms and with PGS (for interaction term models). PGS=polygenic score; PTSD=posttraumatic stress disorder; SE=standard error.

**Supplementary Table 2.** Results of meta-analyses of main effects of polygenic scores and PTSD symptoms, along with their interaction, on blood pressure, stratified by cohort type.

|  | **Systolic Blood Pressure** | | | | | |  | **Diastolic Blood Pressure** | | | | | |
| --- | --- | --- | --- | --- | --- | --- | --- | --- | --- | --- | --- | --- | --- |
| **Effect** | **Beta (SE)** | ***z*** | ***p*-value** | ***Q*** | ***p*-het** | ***I^2^*** |  | **Beta (SE)** | ***z*** | ***p*-value** | ***Q*** | ***p*-het** | ***I^2^*** |
| PGS main effect |  |  |  |  |  |  |  |  |  |  |  |  |  |
| Military cohorts meta-analysis | 1.56 (0.20) | 7.65 | 2.0E-14 | 1.87 | .76 | 0 |  | 0.70 (0.15) | 4.79 | 1.7E-6 | 1.96 | .74 | 0 |
| Community-based cohorts meta-analysis | 3.02 (0.07) | 45.23 | <1E-20 | 5.82 | .05 | 65.7 |  | 1.37 (0.04) | 34.75 | <1E-20 | 1.15 | .56 | 0 |
|  |  |  |  |  |  |  |  |  |  |  |  |  |  |
| PTSD symptoms main effect |  |  |  |  |  |  |  |  |  |  |  |  |  |
| Military cohorts meta-analysis | 4.18 (1.41) | 2.97 | 2.9E-3 | 3.94 | .41 | 0 |  | 1.80 (1.02) | 1.76 | .08 | 0.76 | .94 | 0 |
| Community-based cohorts meta-analysis | -2.18 (0.47) | -4.63 | 3.7E-6 | 1.03 | .60 | 0 |  | 0.64 (0.28) | 2.30 | .02 | 6.68 | .04 | 70.1 |
|  |  |  |  |  |  |  |  |  |  |  |  |  |  |
| PTSD Symptoms x PGS interaction |  |  |  |  |  |  |  |  |  |  |  |  |  |
| Military cohorts meta-analysis | 0.18 (1.40) | 0.13 | .90 | 9.68 | .05 | 58.7 |  | -0.73 (1.01) | -0.73 | .47 | 4.15 | .39 | 3.6 |
| Community-based cohorts meta-analysis | -0.07 (0.46) | -0.16 | .87 | 1.91 | .38 | 0 |  | -0.02 (0.28) | -0.05 | .96 | 4.41 | .11 | 54.7 |

*Note.* Military cohorts included GMRF-QUT, MRS, TRACTS, and VETSA. Community-based cohorts included GTP, SHRS, and UKBB. Models adjusted for the first five ancestry principal components, age, age-squared, sex (for cohorts with men and women), and interactions of all covariates with PTSD symptoms and with PGS (for interaction term models). PGS=polygenic score; p-het=p-value for Cochran’s Q; PTSD=posttraumatic stress disorder; Q=Cochran’s Q; SE=standard error.

**Supplementary Table 3.** Results of meta-analyses of main effects of polygenic scores and PTSD symptoms, along with their interaction, on blood pressure for studies that adjusted for antihypertensive medication use.

|  | **Systolic Blood Pressure** | | | | | |  | **Diastolic Blood Pressure** | | | | | |
| --- | --- | --- | --- | --- | --- | --- | --- | --- | --- | --- | --- | --- | --- |
| **Effect** | **Beta (SE)** | ***z*** | ***p*-value** | ***Q*** | ***p*-het** | ***I^2^*** |  | **Beta (SE)** | ***z*** | ***p*-value** | ***Q*** | ***p*-het** | ***I^2^*** |
| PGS main effect |  |  |  |  |  |  |  |  |  |  |  |  |  |
| Trans-ethnic meta-analysis | 1.45 (0.18) | 8.10 | 5.6E-16 | 6.43 | .60 | 0 |  | 0.78 (0.13) | 6.19 | 6.2E-10 | 12.06 | .15 | 33.7 |
| AA meta-analysis | 1.24 (0.37) | 3.37 | 8.0E-4 | 2.63 | .45 | 0 |  | 0.99 (0.26) | 3.77 | 2.0E-4 | 9.34 | .03 | 67.9 |
| EA meta-analysis | 1.51 (0.20) | 7.39 | 1.5E-13 | 3.40 | .49 | 0 |  | 0.72 (0.14) | 4.99 | 6.1E-7 | 1.92 | .75 | 0 |
|  |  |  |  |  |  |  |  |  |  |  |  |  |  |
| PTSD symptoms main effect |  |  |  |  |  |  |  |  |  |  |  |  |  |
| Trans-ethnic meta-analysis | 2.48 (1.15) | 2.15 | .03 | 9.24 | .32 | 13.4 |  | 0.46 (0.79) | 0.58 | .56 | 8.80 | .36 | 9.1 |
| AA meta-analysis | -0.49 (2.01) | -0.25 | .81 | 1.91 | .59 | 0 |  | -1.46 (1.28) | -1.14 | .26 | 3.90 | .27 | 23.2 |
| EA meta-analysis | 3.95 (1.41) | 2.80 | 5.1E-3 | 4.05 | .40 | 1.2 |  | 1.66 (1.01) | 1.64 | .10 | 1.26 | .87 | 0 |
|  |  |  |  |  |  |  |  |  |  |  |  |  |  |
| PTSD Symptoms x PGS interaction |  |  |  |  |  |  |  |  |  |  |  |  |  |
| Trans-ethnic meta-analysis | 0.05 (1.06) | 0.04 | .97 | 12.44 | .13 | 35.7 |  | -1.27 (0.76) | -1.66 | .10 | 10.94 | .20 | 26.9 |
| AA meta-analysis | 0.26 (1.67) | 0.16 | .88 | 2.33 | .51 | 0 |  | -2.83 (1.21) | -2.33 | .02 | 3.05 | .38 | 1.57 |
| EA meta-analysis | -0.10 (1.37) | -0.07 | .94 | 10.08 | .04 | 60.3 |  | -0.25 (0.98) | -0.26 | .80 | 5.17 | .27 | 22.6 |

*Note.* Studies included were GMRF-QUT, GTP, HOST, MRS, SHRS, TRACTS, and VETSA. Models adjusted for the first five ancestry principal components, age, age-squared, sex (for cohorts with men and women), and interactions of all covariates with PTSD symptoms and with PGS (for interaction term models). AA=African ancestry; EA=European ancestry; PGS=polygenic score; p-het=p-value for Cochran’s Q; PTSD=posttraumatic stress disorder; Q=Cochran’s Q; SE=standard error.

**Supplementary Figure Legends**

**Supplementary Figure 1.** Forest plots of trans-ethnic meta-analyses excluding UK Biobank for the main effect of blood pressure polygenic scores on (A) systolic blood pressure and (B) diastolic blood pressure. For each cohort, a square is plotted at the effect estimate value. The size of each plotted square reflects relative precision, where size is inversely proportional to the standard error of a given cohort. Reported effects are beta coefficients and 95% confidence intervals. AA=African ancestry; EA=European ancestry; FE=fixed effects.

**Supplementary Figure 2.** Forest plots of trans-ethnic meta-analyses excluding UK Biobank for the main effect of posttraumatic stress disorder symptoms on (A) systolic blood pressure and (B) diastolic blood pressure. For each cohort, a square is plotted at the effect estimate value. The size of each plotted square reflects relative precision, where size is inversely proportional to the standard error of a given cohort. Reported effects are beta coefficients and 95% confidence intervals. AA=African ancestry; EA=European ancestry; FE=fixed effects.

**Supplementary Figure 3.** Forest plots of trans-ethnic meta-analyses excluding UK Biobank for the interaction of posttraumatic stress disorder symptoms and blood pressure polygenic scores on (A) systolic blood pressure and (B) diastolic blood pressure. For each cohort, a square is plotted at the effect estimate value. The size of each plotted square reflects relative precision, where size is inversely proportional to the standard error of a given cohort. Reported effects are beta coefficients and 95% confidence intervals. AA=African ancestry; EA=European ancestry; FE=fixed effects.
